# Inhibition of bacteriochlorophyll biosynthesis in the purple phototrophic bacteria *Rhodospirillumrubrum* and *Rhodobacter capsulatus* grown in the presence of a toxic concentration of selenite

**DOI:** 10.1186/s12866-018-1209-5

**Published:** 2018-07-31

**Authors:** Janine Kessi, Stefan Hörtensteiner

**Affiliations:** 1 Institute of Plant Biology, University of Zurich, Zollikerstrasse 107, Zurich, 8008 Switzerland; 2 Kirschenweg 10, Würenlingen, 5303 Switzerland

**Keywords:** Phototrophic *α*-proteobacteria, Selenite reduction, Reactive oxygen species, Se^0^-nanoparticles, Bacteriochlorophyll biosynthesis, Bacteriochlorophyll degradation

## Abstract

**Background** In many works, the chemical composition of bacterially-produced elemental selenium nanoparticles (Se^0^-nanoparticles) was investigated using electron dispersive X-ray analysis. The results suggest that these particles should be associated with organic compounds. However, a complete analysis of their chemical composition is still missing. Aiming at identifying organic compounds associated with the Se^0^-nanoparticles produced by the purple phototrophic bacteria *Rhodospirillum rubrum* and *Rhodobacter capsulatus* (*α* group of the proteobacteria), we used MALDI-TOF spectrometry.

**Results** This technic revealed that numerous signals obtained from particles produced by both species of bacteria were from metabolites of the photosynthetic system. Furthermore, not only bacteriochlorophyll *a*, bacteriopheophytin *a*, and bacteriopheophorbide *a*, which are known to accumulate in stationary phase cultures of these bacteria grown phototrophically in the absence of selenite, were identified. The particles were also associated with intermediary metabolites of the bacteriochlorophyll a biosynthesis pathway such as protoporphyrin IX, protoporphyrin IX monomethyl ester, bacteriochlorophyllide *a* and, most likely, Mg-protoporphyrin IX-monomethyl ester, as well as with oxidation products of the substrates of protochlorophyllide reductase and chlorin reductase.

**Conclusion** Accumulation of intermediary metabolites of the bacteriochlorophyll biosynthesis pathway in these purple phototrophic bacteria was attributed to inhibition of oxygen-sensitive enzymes involved in this pathway. Consistent with this interpretation it has been reported that these bacteria reduce selenite intracellularly, that they contain high levels of glutathione and that the reduction of selenite with glutathione is a very fast reaction accompanied by the production of reactive oxygen species. As many enzymes involved in the biosynthesis of bacteriochlorophyll contain [Fe-S] clusters in their active site, which are known to be degraded in the presence of reactive oxygen species as well as in the presence of molecular oxygen, we concluded that the substrates of these enzymes accumulate in cells during selenite reduction.

Association of metabolites of bacteriochlorophyll biosynthesis and degradation with the Se^0^-nanoparticles produced by *Rhodospirillum rubrum* and *Rhodobacter capsulatus* is proposed to result from coating of the nanoparticles with the intracytoplasmic membrane of these bacteria, where the photochemical apparatus is concentrated.

**Electronic supplementary material:**

The online version of this article (10.1186/s12866-018-1209-5) contains supplementary material, which is available to authorized users.

## Background

Of all the elements, selenium has one of the narrow ranges between dietary deficiency and toxic level [1]. As its geographical distribution on the surface of the earth is largely uneven, some parts contain selenium shortage when toxic levels are present in other parts. Selenium hazards severely affect large population of aquatic and terrestrial life [2]. In natural water selenium concentrations are generally low, but may significantly increase, influenced by anthropogenic and geogenic sources. Diffuse geogenic pollution are widely observed. Accumulation of selenium is observed in various regions of the earth associated with coal, shales, argillaceous sediments and phosphate rocks [3] and references therein. The main aqueous selenium chemical speciation in oxygenated surface water corresponds to selenate and selenite, which are the most bioavailable forms of selenium [4]. These compounds were shown to be highly toxic to most living organisms already at micromolar concentrations [5, 6]. By contrast, many species of bacteria, mostly belonging to the various groups of the proteobacteria, but also firmicute species, were reported to grow in the presence of millimolar concentrations of selenite [7–11]. Selenite-resistant microorganisms were shown to reduce selenite to the much less toxic elemental selenium, and doing so, to produce elemental selenium nanoparticles (Se^0^-nanoparticles). In purple phototrophic bacteria selenite reduction and formation of Se^0^-nanoparticles was reported to occur intracellularly [12, 13]. Analysis of the particles produced by various groups of proteobacteria using electron dispersive X-ray analysis showed signals for carbon and oxygen, indicating that they were associated with organic material [7–13]. In some cases proteins and/or extracellular polymeric substances linked to the particles were identified [14–17]. In the work by Belzile et al. (2006) [17] the authors proposed that Se^0^-nanoparticles produced by *Pseudomonas fluorescens* during selenite reduction were associated with a membrane system.

Altogether these reports indicate that various organic molecules are associated with Se^0^-nanoparticles produced during selenite detoxification in selenite-resistant bacteria. Considering that a detailed knowledge of the chemical composition of these particles should give information about the mechanism(s) of selenite toxicity, we decided to undertake a detailed analysis of these particles. As the metabolism of the purple phototrophic bacteria is well elucidated [18], and as these organisms were shown to survive in the presence of mM levels of selenite [12, 13, 19] and to reduce selenite intracellularly [12, 13], we analyzed the chemical composition of the particles produced by the purple phototrophic bacteria *R. rubrum* and *Rba. capsulatus*.

Purple phototrophic bacteria possess a dense intracytoplasmic membrane system where the photochemical apparatus is concentrated [20], and stationary phase cultures of *Rhodobacter sphaeroides* grown in the absence of selenite, were shown to accumulate bacteriochlorophyll *a* (BChl *a*), bacteriopheophytine *a* (BPhe *a*), bacteriopheophorbide *a* (BPheide *a*), and pyro-bacteriopheophorbide *a* (pyro-BPheide *a*) [21]. In phototrophic prokaryotes pigment accumulation is proposed to result from the absence of pheophorbide *a* oxygenase (PAO), while this enzyme appeared in the course of evolution [22] with the emergence of aerobic photosynthesis. This assumption is consistent with other works reporting that homologs of the genes encoding PAO in higher plants were found in the genomes of unicellular green algae and some cyanobacteria, but not in proteobacteria [23, 24].

Similarly to cultures of *Rba. sphaeroides*, cultures of *Chlamydomonas reinhardtii* grown under aerobic condition accumulated chlorophyll *a* (Chl *a*), pheophorbeide *a* (Pheide *a*) and pyro-pheophorbide *a* (pyro-Pheide *a*) when the cultures were transferred to anaerobiosis at the beginning of stationary phase [25]. It was concluded that activity of PAO was blocked under oxygen limitation and that further degradation of Chl to linear tetrapyrroles did not take place under anaerobic condition.

The results reported by Haidl et al. (1985) [21], and Doi et al. (2001) [25] strongly suggest that stationary phase accumulation of (B)Chl *a*, (B)Phe *a*, (B)Pheide *a* and pyro-(B)Pheide *a* may be a general phenomenon in cultures of phototrophic organisms grown under anaerobic conditions or transferred to anaerobiosis at the time they enter stationary phase.

Concerning the reduction of selenite and the formation of Se^0^-nanoparticles in the purple phototrophic bacteria, it is known that: (i) high levels of glutathione accumulate in the *α*, *β* and *γ* groups of the proteobacteria [26, 27], (ii) the purple phototrophic bacteria reduce selenite intracellularly [12, 13], and (iii) selenite strongly reacts with -SH groups [28–30]. Consequently, glutathione is assumed to function as electron donor in the reduction of selenite in these organisms. This assumption is consistent with previous reports [19, 31] showing that inhibition of glutathione synthesis in cultures of proteobacteria grown in the presence of a toxic concentration of selenite significantly slows down the rate of selenite reduction and decreases the culture growth rate. It is also consistent with the large induction of glutathione reductase in cultures of *Escherichia coli* (*E. coli*) (*γ* group of the proteobacteria) grown in the presence of a toxic concentration of selenite [32].

Furthermore it is known that the reaction of selenite with glutathione produces superoxide anions within the first minutes of the reaction [30, 33] as indicated in Eq. 1.

1 $$ 6 \text{GSH} + 3 \text{SeO}_{3}^{-2} \longrightarrow 3 \text{GS}-\text{Se}-\text{SG} + 2 O_{2}^{-} + 5 H_{2}O  $$

This reaction implies that intracellular reduction of selenite in glutathione-containing cells is accompanied by the formation of superoxide anions $\left (O_{2}^{-}\right)$, which are transformed to hydrogen peroxide and molecular oxygen in the presence of superoxide dismutase(s) (SOD(s)), and that hydrogen peroxide will be degraded to water and molecular oxygen in the presence of catalases.

Consistent with the involvement of glutathione in the reduction of selenite in proteobacteria, is the large induction of SOD and catalase activities measured in *E. coli* exposed to selenite [32]. It also agrees with the observation that hypersensitivity of a mutant of *E. coli* lacking both SodA and SodB activities was relieved in a triple mutant lacking, in addition, glutathione synthesis activity, indicating that the absence of glutathione relieves the hypersensitivity of strains lacking SOD’s [32]. It furthermore agrees with the reported enhanced viability, in the presence of selenite, of a strain of *Salmonella typhimurium* (*γ*-group of the proteobacteria) which overexpresses proteins involved in protecting cells from oxidative stress [34]. Therefore the effect of oxidative stress can be expected to be revealed in the metabolism of purple phototrophic bacteria grown in the presence of a toxic concentration of selenite.

In the present work we observed that the substrate or substrate derivative of many enzymes involved in BChl *a* biosynthesis accumulated in cells of *R. rubrum* and *Rba. capsulatus* grown in the presence of 0.5 mM selenite. This observation led to the hypothesis that accumulation of these metabolites resulted from the oxidative stress the cells undergo in the presence of selenite, leading to inhibition of the respective enzymes. Indeed, several enzymes involved in the BChl biosynthesis pathway contain [Fe-S] clusters at their active site, which were shown to be very unstable in the presence of molecular oxygen and ROS such as superoxide anions and hydrogen peroxide [35–38]. Interestingly, these intermediary metabolites of BChl biosynthesis were associated with the Se^0^-nanoparticles these organisms excreted as a final step of selenite detoxification.

Identification of these compounds was performed using mass spectrometry complemented with demetalation experiments. Demetalation allowed identifying more accurately several MS-signals. Aiming at testing the stability of the particle structure, and at ensuring that the obtained mass spectrometry signals (MS-signals) represented molecules which were bound to the particles, and not to membrane vesicles (MVs) which contaminated the Se^0^-nanoparticles samples, MS-analysis was performed on native as well as on detergent-washed particles.

## Methods

### Reagents and bacterial strains

All organic solvents used were analytical reagent grade or HPLC grade and were used without further purification. Water was purified using a milliQ purification device (Millipore, Merck, Switzerland).

Selenium(IV) oxide 98%, the reference compounds protoporphyrin IX (PPIX), protoporphyrin IX dimethyl ester (PPIX-di-O- *CH*_3_), as well as BChl *a*_*p*_ (BChl *a* esterified with the isoprenoid alcohol phytol) from *Rba. sphaeroides* were from Sigma-Aldrich (Buchs, Switzerland). Diheptanoyl-phosphatidylcholine (DHPC) was from Avanti Polar Lipids (Alabaster, Alabama). The matrices 2,5-dihydroxybenzoic acid (DHB), and 2,4,6-trihydroxyacetophenone (THAP), were from Sigma-Aldrich (Buchs, Switzerland).

Selenium(IV) oxide was prepared as a 20 mM solution in degassed, sterile culture medium. DHPC was prepared as a 25 mM solution in 20 mM Na ^+^-,K ^+^-phosphate buffer, pH 7.2. The reference molecules were dissolved in methanol at a concentration of 1.5 mg/ml.

The bacterial strains used were *R. rubrum* S1 (DSM 467) and *Rba. capsulatus* B10 (ATCC 33303).

### Growth of bacteria

Bacteria were grown under phototrophic anaerobic condition in a Sistrom minimal medium under incandescent light, as described previously [12] in 100 ml or 500 ml flasks closed with airtight rubber stoppers fixed with a metal ring. Selenite (selenium(IV) oxide solution) thoroughly purged with N _2_ gas to eliminate molecular oxygen, was added to a final concentration of 0.5 mM, using a syringe which was also purged with N _2_ gas, at the time the cultures were transferred to light.

### Isolation of the Se ^0^-nanoparticles

Cultures grown in the presence of a toxic concentration of selenite (0.5 mM) were harvested two days after the end of selenite reduction, i.e. two days after exhaustion of selenite from the culture medium. Selenite concentration was measured spectrophotometrically in the form of a complex with diaminonaphtalene as previously described [12]. Cells were centrifuged at 10 000 g at room temperature. The cell supernatant was centrifuged once more under the same condition in order to eliminate possible contaminating cells. The supernatant containing the particles was cooled down on ice and centrifuged at 100 000 g for 40 min at 4 °C. The sedimented particles were resuspended in a volume of ice-cold Na^+^-, K^+^ phosphate buffer, 20 mM, pH 7.2, corresponding to about 1/10 of the initial culture volume. Since after centrifugation a large part of the particles was attached to the wall of the centrifugation tube, resuspension was done with help of an ultrasonic homogenizer (Bandelin HD 2070; Sigma-Aldrich, Switzerland). The device was set at 40% amplitude and a pulsation mode of 1 sec with 50% pulse time. Five to ten pulses were applied and the particles were centrifuged once more as indicated above. This washing procedure was repeated twice. Finally, the particles were resuspended as indicated above but in a volume of ice-cold phosphate buffer corresponding to about 1/40 of the initial culture volume. They were used immediately for analysis or frozen as 0.5 ml samples in liquid nitrogen and stored at -80 °C. These particle samples are designated as “native particles samples” in the following. Se^0^-concentration varied between 1.0 and 2.0 *μ*moles/ml in these samples.

Isolation of the Se ^0^-nanoparticles from the culture medium and not from the cells ensured that they were as little as possible contaminated by metabolites contained within the cells.

### Determination of elemental selenium

Se^0^ was oxidized to selenite using concentrated nitric acid as previously described [30]. According to the report of Waitkins and Clark (and references therein) [39] this method converts elemental selenium to selenium dioxide with a yield greater than 95% in the presence of an excess of nitric acid. Selenite was then determined spectrophotometrically in the form of a complex with diaminonaphthalene as previously described [12].

### Transmission electron microscopy (TEM)

A droplet of native particle suspension was placed on a formvar, glow discharged TEM grid, and the particles were left to sediment for 1 min. Afterwards, excess liquid was wicked off using filter paper, the sample was left drying for 20 min in a Petri dish and the particles were analyzed without any additional treatment in a CM12 transmission electron microscope (FEI, Eindhoven, The Nethertland) at 120 kV acceleration voltage.

### Washing Se^0^-nanoparticles with detergent

An ice-cold sample of a native particle suspension obtained as indicated above was rapidly mixed with the same volume of ice-cold 20 mM Na^+^-,K^+^-phosphate buffer, pH 7.2, containing 25 mM of the detergent DHPC. This detergent was shown to efficiently solubilize various biological membranes [40]. It was therefore considered to also efficiently solubilize the membrane vesicles (MVs), which were contaminating the Se^0^-nanoparticle samples. The suspension was rapidly mixed by repeated pipetting, incubated on ice for 15 min with mixing every 2 min, and then centrifuged at 100 000 g and 4 °C for 40 min. Afterwards, the particles were washed once with the two-fold original volume of ice-cold phosphate buffer without detergent, and twice with ice-cold milliQ water. Finally, the particles were resuspended in the initial sample volume of milliQ water, frozen as 0.5 ml samples in liquid nitrogen, and stored at -80 °C. These particles are designed as “detergent-washed particles” in the following. After each centrifugation, the particles were resuspended with help of an ultrasonic homogenizer as indicated above.

### Preparation of Se^0^-particle extracts

Extraction of the particle samples was performed using a described method [41]. However, in order to maximize preservation of chemical structures, and particularly, to avoid oxidation of extracted molecules [42, 43] or hydrolysis of lipids [44], extraction was performed at dim light, at a temperature close to 0 °C, and under slightly acid condition using the following protocol:

HCl was added to a final concentration of 1.5 mM to 1.0 volume of native Se ^0^-nanoparticle samples. This yielded a pH close to 4 in the particle samples. Then, 3.7 volumes of a mixture of ice-cold CHCl _3_/MeOH (1:2, v/v) were added to the suspension, and the sample was thoroughly and rapidly mixed, giving a particle suspension in a monophasic CHCl _3_/MeOH/H _2_O extraction mixture. The sample was incubated on ice for 15 min and then centrifuged for 20 min at 20 000 g and 4 °C. The supernatant was transfered to a new tube, taking care that the entire particle content remained in the pellet. The particle pellet was replenished with ice-cold milliQ water to the initial sample volume, the particles were resuspended by sonication as indicated above, and extraction was repeated in the same way. Three extractions were performed. Separation of the CHCl _3_/MeOH/H _2_O extract into two phases was forced by addition of 200 *μ*l of CHCl _3_ when necessary. The water phases were twice re-extracted with cold CHCl _3_, and the combined organic phases evaporated under a stream of N _2_ gas. The small residue was dissolved in 20-30 µl of CHCl _3_/MeOH (1:2, v/v) and stored at -20 °C.

### Demetalation of Mg-binding molecules in particle sample extracts

Tetrapyrrole pigments complexed with Mg were demetalated using concentrated acetic acid (Prof. H. Scheer, Univ. München, personal communication). One drop of concentrated acetic acid was added to 20 µl (30 µg) of a stirred solution of the reference BChl *a*_*p*_ or to 20 µl of a stirred solution of a concentrated particle sample extract obtained from native particles, and the reaction was allowed to take place for 1 min. The acetic acid was then evaporated under a stream of N _2_ gas, and the residue was dissolved in 20 µl of CHCl _3_/MeOH (1:2, v/v) and stored at -20 °C. The MS-spectra of the particle sample extract before and after demetalation were compared.

To analyze whether other metals (for example Fe) were present in the particle samples and formed a more stable metal-porphyrin complex or metal-bacteriochlorin complex than Mg [45] in the analyzed samples, a drop of concentrated HCl saturated with FeSO _4_ [46] was added to the sample spot of a particle extract already treated with concentrated acetic acid, and the spot was dried under a stream of N _2_ gas.

**Table 1:** Relative intensities of MS-signals from native and detergent-washed Se^0^-nanoparticles produced by *R. rubrum* and *Rba. capsulatus* identified according to the mass values obtained in the MS-spectra

	

Intensities are indicated as approximative percent of the base peak ( = 100%). Each intensity value represents the mean of three measurements. The measurements were performed with samples embedded in the DHB-matrix

As the sample spots were not completely homogeneous and as desorption-ionization was most likely not equally efficient for each analyte, it was not possible to exactly determine the ratio of demetalated to non-demetalated compounds. Indications of demetalation efficiency were calculated by normalizing the MS-signal intensity obtained for the non-demetalated compound to that obtained for the respective demetalated compound in each spectrum (before and after treatment with conc. acetic acid). The normalized intensity after acid treatment was then expressed as percent of the normalized intensity before acid treatment (Table 2).

**Table 2:** Demetalation experiments

Samples	Initial mass (m/z)	Final mass (m/z)	Decrease of initial mass (m. u.)	*σ*(%)
Reference BChl *a*_p_	910.5	888.6	21.9	9.3
Reference Bchlide *a*	632.3	610.3	22	2.3
Particle sample extracts *R. rubrum*	905.5	882.6	21.9	**
Particle sample extracts *R. rubrum*	632.3	610.3	22.0	4.5
Particle sample extracts *Rba. capsulatus*	616.2	616.2	–	93.5
Particle sample extracts *Rba. capsulatus*	595.3	595.3	–	91.6

Signal intensities were measured before (Initial mass) and after treatment (Final mass) of reference molecules or particle sample extracts with conc. acetic acid (see “Methods section”). *σ* represents the final signal intensity of the metalated compound expressed in % of its initial intensity (see text).

^**^A signal of high intensity was obtained at m/z 882.6 in the MS-spectrum of this particle sample extract already before addition of concentrated acetic acid. This may indicate that Bchl *a*_g_ was largely demetalated during the extraction process (Additional file 6) and, most likely, that BPhe *a*_g_ was more efficiently detected in the particle sample extracts than in the native particle samples. Consequently, demetalation was difficult to quantify for this molecule

As MS-signals at m/z 595.3 and m/z 616.2 did not yield any increased signal corresponding to the loss of Mg after acid treatment, they were normalized to the stable signal for PPIX-O-CH _3_ at m/z 577.3 in both the non-treated and the acetic acid-treated samples.

### MALDI-TOF mass spectrometry

Matrix compounds THAP (pKa 6.3) and DHB (pKa 3.0) were dissolved (freshly each day) in acetonitrile/water (7:3, v/v) at 10 mg/ml. Samples were prepared by first spotting 0.6 *μ*l matrix solution onto the MALDI target and allowing the spot to dry at room temperature. 0.3 *μ*l particle sample, particle extract or reference compound solution was then applied to the matrix spot before re-drying as above.

The mass spectrometry analyses were performed with a 4800 Plus MALDI TOF/TOF instrument (AB SCIEX, Framingham, MA, USA) in the positive ion mode. The system utilizes 200 Hz Nd:YAG laser emitting at 355 nm. The extraction voltage was 20 keV. In the MS reflector mode, 1000 sub-spectra were accumulated, typically in the mass range from 300-3000 mass units (m. u.).

The following parameter settings were applied in the MS/MS mode: collision energy: 1 kV, collision gas pressure: 2.5·10^-6^ Torr, timed ion selector resolution: 200, accumulation of 2000 sub-spectra.

The reflector mode of MALDI-TOF/TOF mass spectrometer was calibrated by employing a mixture of known peptides (Cal Mix, AB Sciex) with masses in the 900 to 2100 mass range. Upon calibration of the spectra acquired from the standard peptide mixtures, a plate calibration was performed for all positions on the MALDI target plate. The MS/MS mode calibration was based on a fragment ion spectrum obtained from Glu-fibrinopeptide B (m/z 1570).

Four thousand Series Explorer Software (AB Sciex) was used for instrument control and acquisition of spectra, and PeakView Software (AB Sciex) was applied to data analysis.

### Standard errors

#### MS-spectra

For each bacterial species MS-spectra of native as well as of detergent-washed particle samples obtained from 3 different cultures were recorded. Standard errors on the intensity of the MS-signals were in the range of 0.1 to 5.2% for most signals, except for the signals at m/z 563.3 and m/z 632.2 obtained in the MS-spectra of the native particle samples isolated from cultures of *R. rubrum*, which yielded standard errors of 8.4 and 14.7%, respectively.

#### Demetalation

For each bacterial species demetalation was performed on 2 different extracts obtained from different Se^0^-nanoparticle samples.

Demetalation results are the mean of two to three experiments. Standard errors were comprised between 1.8 and 4.0% for most measurements, but the MS-signals at m/z 595.3 and m/z 616.2 yielded errors of 8.4 and 6.5%, respectively.

#### Tandem MS-spectra

Signal intensities of product ions in the schematic representation of tandem MS-spectra are the mean of two to three measurements in the cases of BChl and BPhe (Table 3) and the mean of three to five measurements in the cases of porphyrin and bacteriochlorin derivatives (Figs. 4, 5, 6, 7 and 8). Each set of measurements represented particle samples from at least two different bacterial cultures. Standard errors were comprised between 0.0 and 5.0% for most measurements, but a maximal standard error of 8.4% was obtained in the tandem MS-spectra of porphyrin and bacteriochlorin derivatives.

**Fig. 1:**
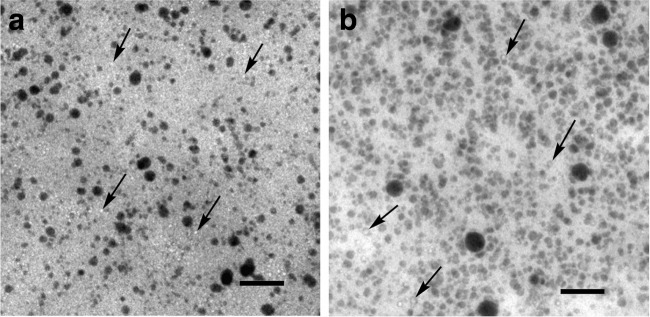
Electron micrographs of native Se^0^-nanoparticle samples. **a**) Particle samples obtained from the culture medium of *R. rubrum*. **b**) Particle samples obtained from the culture medium of *Rba. capsulatus*. The particle samples were deposited on a TEM grid and observed without any treatment. Bars = 200 nm

**Fig. 2:**
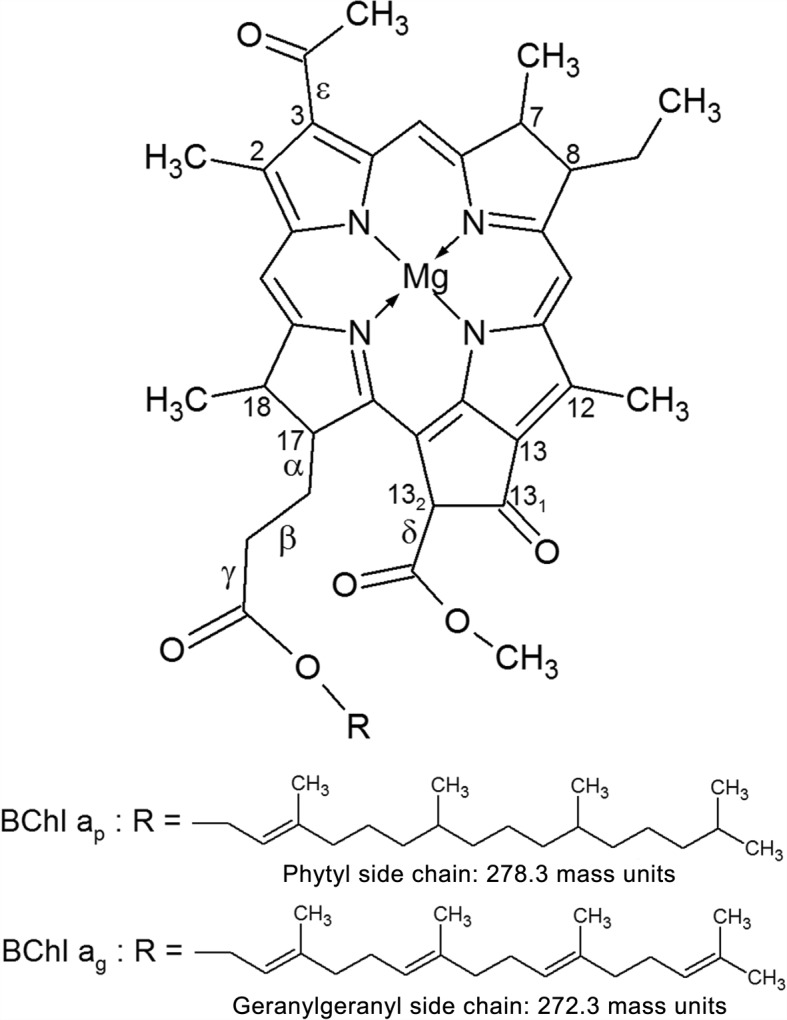
Structure of BChl *a* and of some of its derivatives. 1: BPhe *a*_p_ : R = phytyl and Mg^++^ replaced by 2H^+^ 2: BPhe *a*_g_ : R = geranylgeranyl and Mg^++^ replaced by 2H^+^ 3: BChlide *a* : R = H 4: BPheide *a* : R = H and Mg^++^ replaced by 2H^+^. The carbon atoms were numbered according to the IUPAC (International Union of Pure and Applied Chemistry) nomenclature

**Fig. 3:**
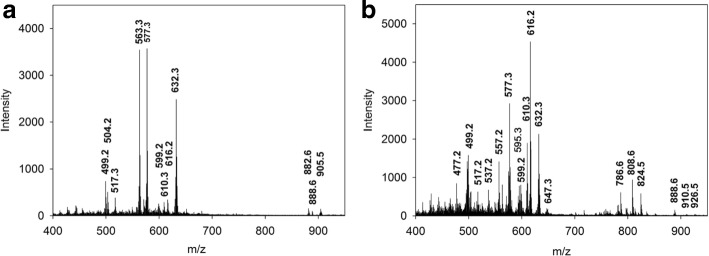
Representative MS-spectra of native Se^0^-nanoparticle samples. Particle samples prepared using the DHB-matrix. **a**) Particle samples isolated from the culture medium of *R. rubrum*. **b**) Particle samples isolated from the culture medium of *Rba. capsulatus*

**Fig. 4:**
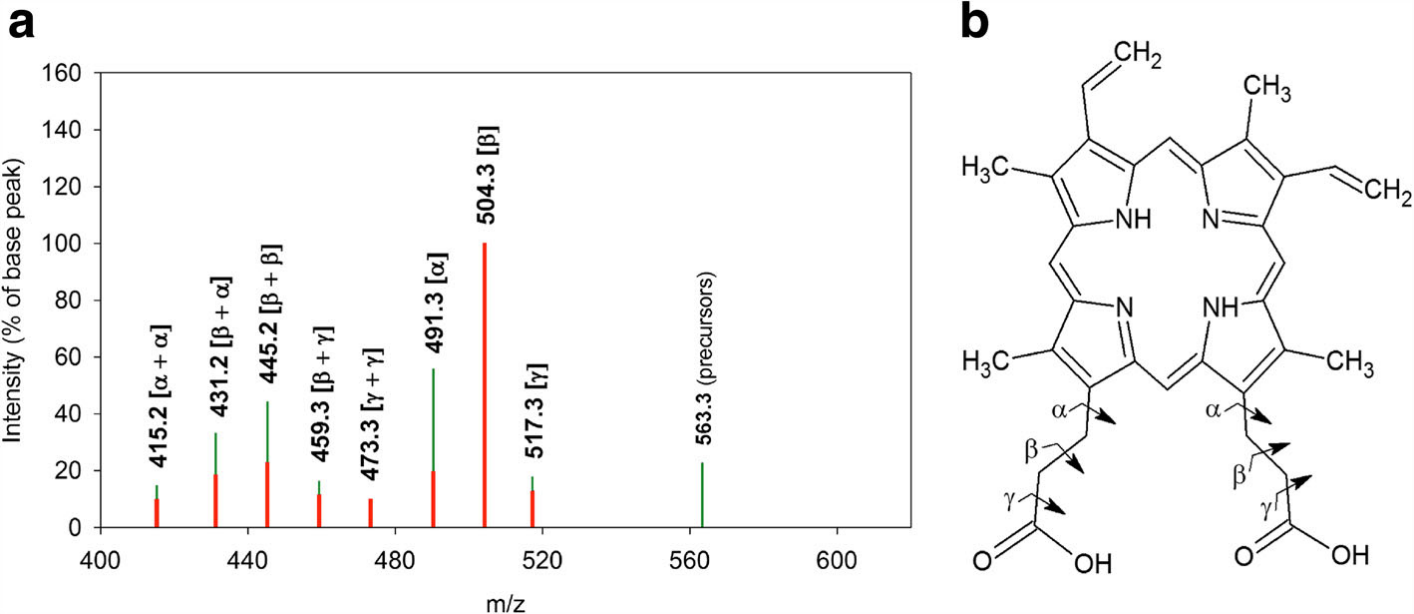
Schematic representation of the tandem MS-spectrum of the signal at m/z 563.3. **a**: Spectrum of the signal obtained from the Se^0^-nanoparticle samples isolated from cultures of *R. rubrum*: Red bars. Comparison with the spectrum of the reference PPIX: Green bars. The bond cleavage(s) corresponding to the fragment loss(es) are indicated in parentheses on top of each bar representing molecular ions obtained in the MS/MS-spectra of both MS-signals at m/z 563.3. **b**: Structure of PPIX with indication of bond cleavage locations

**Fig. 5:**
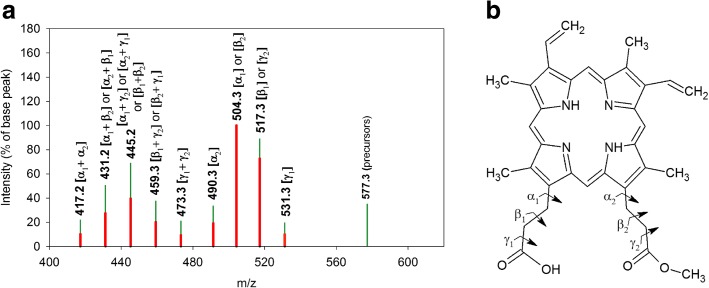
Schematic representation of the tandem MS-spectrum of the signal at m/z 577.3. **a**: Spectrum of the signal obtained from the Se^0^-nanoparticle samples isolated from cultures of *R. rubrum*: Red bars. Comparison with the spectrum of the reference PPIX-O-CH_3_: Green bars. The bond cleavage(s) corresponding to the fragment loss(es) are indicated in parentheses on top of each bar representing molecular ions obtained in the MS/MS-spectra of both MS-signals at m/z 577.3. **b**: Structure of PPIX-O-CH_3_ with indication of bond cleavage locations

**Fig. 6:**
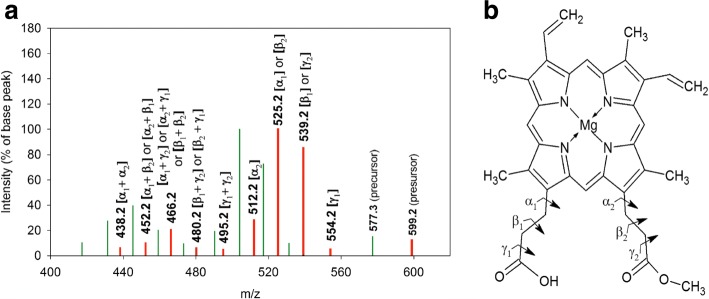
Schematic representation of the tandem MS-spectrum of the signal at m/z 599.3/598.3. **a**: Spectrum of the signal obtained from the Se^0^-nanoparticle samples isolated from cultures of *R. rubrum*: Red bars. Comparison with the spectrum of the reference PPIX-O-CH_3_: Green bars. The bond cleavage(s) corresponding to the fragment loss(es) are indicated in parentheses on top of each bar representing the molecular ions obtained in these MS/MS-spectra. **b**: Proposed structure for the molecule yielding the MS-signal at m/z 599.3 or m/z 598.3 with indication of bond cleavage locations. It is based on: 1: The absence of this signal in acid particle sample extracts (demetalation). 2: The high fragmentation similarity between the analyte and PPIX-O-CH_3_ yielding a mass difference of 21 or 22 m. u. between each molecular ion (Table 4)

**Fig. 7:**
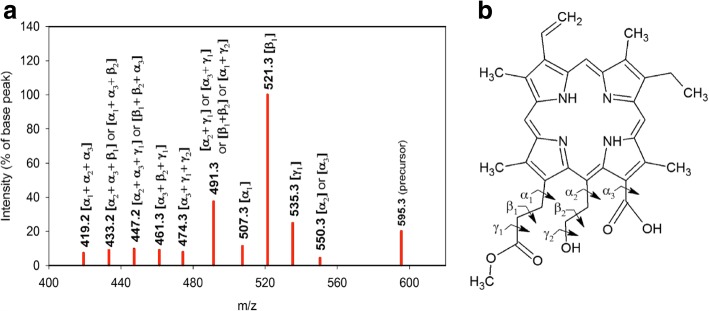
Schematic representation of the tandem MS-spectrum of the signal at m/z 595.3. **a**: Spectrum of the signal obtained from the Se^0^-nanoparticle samples isolated from cultures of *Rba. capulatus*. The bond cleavage(s) corresponding to the fragment loss(es) are indicated in parentheses on top of each bar representing molecular ions obtained in these MS/MS-spectra. No reference representing a similar compound was available. **b**: Proposed structure for the molecule yielding this MS-signal with indication of bond cleavage locations. It is base on: 1. The results of demetalation showing that this MS-signal represented a metal-free compound. 2. The low difference between the measured mass (595.302 m. u.) and the theoretical mass (595.284) of the proposed structure. 3. The absence of signal for the loss of an acetoxy group [-CO-CH_3_], indicating that it represented a metabolite of BChl biosynthesis containing a C3-vinyl group. 4. The large number of double fragment losses corresponding to the loss of 104 m. u. (m/z 491.3) and the large number of signals proposed to represent the loss of three fragments. These results were consistent with the presence of substituents bound not only to the C13 and C17 carbon atoms, but also to the C15 carbon atom of the tetrapyrrole ring, thus representing a porphyrin instead of a bacteriochlorin molecule. 5. The odd m/z value obtained for this signal, which corresponds to a protonated molecular ion as obtained for the references PPIX (m/z 563.3) and PPIX-O-CH_3_ (m/z 577.3), and is consistent with the proposed porphyrin structure

**Fig. 8:**
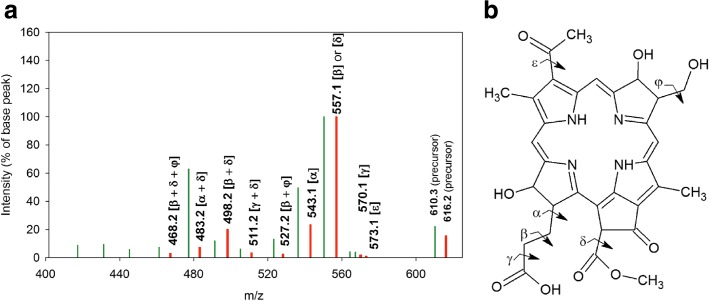
Schematic representation of the tandem MS-spectrum of the signal at m/z 616.2. **a**: Spectrum of the signal obtained from the Se^0^-nanoparticle samples isolated from cultures of *Rba. capulatus*: Red bars. Comparison with the spectrum of the reference BPheide a: Green bars. The bond cleavage(s) corresponding to the fragment loss(es) are indicated in parentheses on top of each bar representing molecular ions obtained in these MS-spectra. **b**: Proposed structure for the molecule yielding this MS-signal with indication of bond cleavage locations. It is based on: 1. The results of demetalation showing that it represented a metal-free molecule. 2. Similarities between its MS/MS fragmentation pattern with that of BPheide *a* (see Table 5). 3. The low difference between the measured mass (616.212 m. u.) and the theoretical mass (616.217 m. u.) of the proposed structure. 4. The low intensity of most of the tandem MS-signals of m/z 616.2 compared to the corresponding signals obtained for the referemce BPheide *a*. This effect is proposed to result from the extension of the bacteriochlorine *π*-resonance system to the C7-O and C18-O bonds of m/z 616.2, leading to a lower flexibility of the molecule. 5. The low fractional part of the molecular mass of this MS-signal compared to that of BPheide *a*, indicating that it contains a relatively large number of oxygen atoms and a relatively low number of hydrogen atoms (see text)

**Table 3:** Fragmentation patterns of signals at m/z 904.5, m/z 905.5, m/z 888.6 and m/z 882.6 obtained in the MS-spectra of Se°-nanoparticle samples and of particle sample extracts

Analytes ^∗^	Precursor ion m/z	Product ions							
		1^st^		2^nd^		3^rd^		4^th^	
		m/z	Intensity (% max.)	m/z	Intensity (% max.)	m/z	Intensity (% max.)	m/z	Intensity (% max.)
BChl *a*_p_ Reference	910.5	632.2	100	—	—	—	—	—	—
BChl *a*_g_ Particle samples *R. rubrum*	904.5	632.2	100	572.2	6.4	—	—	499.2	12.2
Bphe *a*_p_ Reference	888.6	610.3	100	549.3	28.1	537.3	11.1	477.3	14.1
Bphe *a*_p_ Particle sample extracts *Rba. capsulatus*	888.6	610.3	100	549.3	22.1	537.3	16.7	477.3	14.3
Bphe *a*_p_. Particle sample extracts *R. rubrum*	888.6	610.3	100	550.3	15.5	537.3	12.6	477.3	10.9
Bphe *a*_g_. Particle samples *R. rubrum*	882.6	610.3	100	551.3	12.1	537.3	16.9	477.3	13.6
Bphe *a*_g_. Particle sample extracts *R. rubrum*	882.6	610.3	100	549.3	16.8	537.3	12.0	477.3	14.7

Tandem MS-spectra of MS-signals obtained for particle samples or particle sample extracts were compared with those of the references BChl *a*_p_ and BPhe *a*_p_. Note that in each MS/MS-spectrum the most intense signal was for the loss of a 278.3 m. u. fragment or of a 272.3 m. u. fragment. As the mass of these fragments exactly corresponded to that of the phytyl side chain or the geranylgeranyl side chain, respectively (Fig. 2), these results confirmed that each of these MS-signals represented a BChl (BChl *a*_p_ or BChl *a*_g_) or a BPhe (BPhe *a*_p_ or BPhe *a*_g_) molecule.

^*^Identified according to the mass values obtained in the MS-spectra

**Table 4:** Fragmentation pattern of the signal at m/z 599.2/598.2 obtained in the MS-spectrum of Se^0^-nanoparticle samples isolated from cultures of *R. rubrum*

Product ions (m/z)		*Δ* M (m. u.)	Signal intensity (% of base peak)	
PPIX-O-CH_3_ particle samples	m/z 599.2 particle samples	m/z 599 - PPIX-O-CH_3_	PPIX-O-CH_3_ particle samples	m/z 599.2 particle samples
531.3	**553.2**	22	9.8	**5.5**
517.3	**539.2**	22	72.6	**85.6**
504.3	**525.2**	21	100.0	**100.0**
490.3	**511.2**	21	19.3	**28.3**
473.3	**495.2**	22	9.4	**5.1**
459.3	**481.2**	22	20.2	**6.4**
445.3	**466.2**	21	39.6	**20.9**
431.3	**452.2**	21	27.5	**10.4**
417.3	**438.2**	21	10.2	**6.4**

The fragmentation pattern of the MS-Signal at m/z 599.2/598.2 (bold typing) was compared with that of PPIX-O-CH_3_ (m/z 577.3) identified in the same particle samples (normal typing). Note that: 1) Each signal obtained in the MS/MS-spectrum of the analyte and of PPIX-O-CH_3_ was for the same fragment loss, yielding a constant mass difference of 21 m. u. or 22 m. u. between each respective product ion of analyte and PPIX-O-CH_3_. 2) This mass difference well agreed with the mass of Mg which was replaced by two or three protons. This result strongly suggested that the analyte corresponded to Mg-PPIX-O-CH_3_

**Table 5:** Fragmentation pattern of the signal at m/z 616.2 obtained in the MS-spectrum of Se^0^-nanoparticle samples isolated from cultures of *Rba. capsulatus*

Product ions (m/z)		*Δ*M (m. u.)	Signal intensity (% of base peak)	
BPheide *a* reference	m/z 616.2 particle samples	m/z 616.2 - BPheide *a*	BPheide *a* reference	m/z 616.2 particle samples
567.3	**573.1**	**6**	8.8	**1.0**
564.3	**570.1**	**6**	9.6	**2.0**
551.3	**557.1**	**6**	100.0	**100.0**
537.3	**543.1**	**6**	21.7	**23.4**
521.3	**527.2**	**6**	9.1	**2.4**
505.3	**511.1**	**6**	8.4	**3.4**
491.3	**498.2**	**7**	18.6	**20.0**
477.3	**483.2**	**6**	23.8	**4.8**
461.3	**468.2**	**7**	5.9	**2.9**
445.2	**—**	**—**	5.2	**—**
431.2	**—**	**—**	9.0	**—**
417.2	**—**	**—**	9.7	**—**

The fragmentation pattern of the MS-signal at m/z 616.2 (bold typing) was compared with that of the reference BPheide *a* (normal typing). Note that most signals obtained in the MS/MS spectrum of analyte and reference BPheide *a* was for the same fragment loss (± 1 m. u.), strongly suggesting a high structure similarity between analyte and BPheide *a*

## Results

### Transmission electron microscopy

TEM pictures of native Se^0^-nanoparticle samples are presented in Fig. 1. The particles from both bacterial species were nearly spherical. The diameter of the native particles isolated from the cultures of *R. rubrum* varied between 10 nm and 90 nm, with the majority having a diameter between 15 nm and 30 nm. The diameter of most of the native particles isolated from cultures of *Rba. capsulatus* was between 25 nm and 40 nm, while a small number of them had a diameter of 80 nm to 90 nm.

Together with electron dense Se^0^-nanoparticles, electron micrographs showed small transparent MVs (see arrows in Fig. 1). A large number of very small MVs was present in particle samples prepared from cultures of *R. rubrum* (Fig. 1a). MVs were significantly less abundant in particle samples obtained from cultures of *Rba. capsulatus* (Fig. 1b), where a ratio of MVs to nanoparticles of approximately 1:40 could be counted on the TEM pictures (500 nanoparticles).

### MS-spectra

#### Matrix effect on reference molecules and molecules contained in particle samples

**Reference molecules.**

In a first approach we tested the performances of the DHB- and of the THAP-matrices for MS-analysis of reference molecules. In the DHB-matrix the reference BPhe *a* yielded an intense signal (m/z 888.6) indicating a good stability of this molecule under the measurement condition used (Additional file 1A). In contrast, the reference BChl *a* (m/z 910.5/911.5) was partially demetalated and dephytylated when embedded in this matrix, thus yielding additional signals for BPhe *a*_*p*_ (m/z 888.6) and BChlide *a* (m/z 632.2) as well as for supplementary BChl *a*_*p*_ degradation products (Additional file 1B). This result was expected, as demetalation (pheophytinization) of Chl or BChl increases with decreasing pH, with a particular increase below pH 3.5 [47], and cleavage of the phytylester bond is promoted by laser light absorption of the pigment [48]. By contrast, and owing to the neutral pKa of the THAP-matrix, the chemical structures of both BChl *a*_*p*_ and BPhe *a*_*p*_ were well preserved in this matrix (Additional file 2).

A schematic representation of BChl *a* and of some of its derivatives is presented in Fig. 2.

**Molecules contained in particle samples.**

Samples of Se^0^-nanoparticles produced by both *R. rubrum* and *Rba. capsulatus*, and embedded in the DHB-matrix, showed various MS-signals with m/z values corresponding to those obtained for the reference molecules BChl *a*_*p*_ and PPIX and to known derivatives of reference molecules such as PPIX-O-CH _3_, BChlide *a* or BPheide *a* (see below). By contrast, MS-spectra from samples prepared with the THAP-matrix, particularly from the particle samples obtained from cultures of *R. rubrum*, were dominated by unidentified signals, while signals representing BChl *a* and its derivatives were weak or even absent (Additional file 3).

Considering that many of the MS-signals we obtained were suspected to represent metabolites of BChl biosynthesis and degradation (see below) we focused our analysis on the impact of selenite stress on BChl metabolism. We therefore mostly measured samples embedded in the DHB-matrix.

#### Ionic forms of the analytes

As the first and second protonation steps of pyro-Phe *a* in methanolic hydrochloric acid were reported to take place at pH 4.14 and pH 2.06, respectively [49], the pH of the samples analyzed in this work, combined with the acid pKa of the DHB-matrix, led to a pH of the sample spots close to the pH of the first protonation step. Consequently, both the protonated molecular ions [M + H]^+^ and the molecular ion radicals [M^**.**^]^+^ were expected to occur in the MS-spectra.

In our experiments signals attributed to porphyrin molecules such as PPIX, PPIX-di-O-CH _3_, and PPIX-O-CH _3_ yielded protonated molecular ions with odd mass unit values. By contrast, signals attributed to bacteriochlorin derivatives such as BPhe *a*, BChlide *a*, and BPheide *a* most often appeared as molecular ion radicals with even mass unit values (Table 1). Signals attributed to BChl *a* molecules, as well as to porphyrin, which were assumed to contain Mg (Mg-PPIX-O-CH _3_), appeared as protonated molecular ions or molecular ion radicals, depending on the initial pH of the sample.

#### MS-spectra of compounds associated with native Se ^0^-nanoparticles contaminated by MVs

Representative MS-spectra of native Se ^0^-nanoparticle samples obtained from cultures of *R. rubrum* and *Rba. capsulatus* prepared with the DHB-matrix are presented in Fig. 3. The various MS-signals were provisionally identified according to the mass values obtained in the MS-spectra. Mass and intensity of these MS-signals are listed in Table 1. It must be noted that the intensity of the signals representing BChl *a*_*p*_ and BChl *a*_*g*_ (BChl *a* containing a geranyl-geranyl moiety) were underestimated due to the instability of these compounds when embedded in the acid DHB-matrix (Additional file 1B). As the intensity of these MS-signals was particularly low (0.92 and 0.99% of the maximal measured intensities, respectively), contribution of the degradation products for BPhe *a*, BChlide *a* and BPheide *a* can be considered to be negligible, i.e. in the range of the measurement error.

As already mentioned, these spectra showed many signals which corresponded to m/z values of molecular ion radicals or protonated molecular ions of reference molecules or assumed derivatives of reference molecules. The MS-signal at m/z 616.2 was attributed to 3-vinyl BChlide or protoheme according to literature data [50, 51]. A more reliable identification of these various MS-signals was however obtained by analysis of their tandem mass spectra complemented with results of demetalation experiments (see below).

#### MS-spectra of compounds associated with detergent-washed Se ^0^-nanoparticles.

In order to control whether the MS-signals obtained for the Se^0^-nanoparticle samples really belonged to the particles and not to the MVs, which were contaminating the particles, the particle samples were washed with the detergent DHPC, which was shown to efficiently solubilize various biological membranes [40].

Interestingly the detergent-washed Se^0^-nanoparticles were associated with the same molecules as the native particle samples, except for the MS-signals with m/z values larger than m/z 632.2, which mostly disappeared from the MS-spectra after the detergent-washing procedure (Table 1 and Additional file 4). This result suggested that the compounds represented by the MS-signals with m/z values smaller than or equal to m/z 632 were really associated with the particles, and that they were even tightly bound to them.

Concerning the compounds represented by MS-signals larger than m/z 632.2, we propose that they were, initially, likely also associated with the Se^0^-nanoparticles but, according to their hydrophobic character, i.e. their strong affinity for the detergent, were released from the particles during the washing procedure and incorporated in the mixed detergent-membrane micelles.

Most MS-signals listed in Table 1 and yielding m/z values comprised between 905.5 and 563.3 were identified using demetalation and/or comparison of their tandem MS-spectra with that of reference molecules. For identification of the signals at m/z 824.6, m/z 808.6 and m/z 786.6, obtained in the MS-spectra of the Se^0^-nanoparticle samples isolated from cultures of *Rba. capsulatus*, see Additional file 5.

### Demetalation experiments

#### Demetalation of the references BChl *a*_*p*_ and BChlide *a*

Demetalation results are presented in Table 2. After treatment with conc. acetic acid (see “Methods section”) the intensity of the MS-signal at m/z 911.5/910.5, representing the reference BChl *a*_*p*_, decreased to 9.3% of its original intensity, while the signal at m/z 632.2 representing dephytylated BChl *a*_*p*_, i.e. BChlide *a* (see Fig. 2), decreased to 2.3% of its initial intensity. They were replaced by signals for BPhe *a*_*p*_ and BPheide *a* at m/z 888.6 and m/z 610.3, respectively (see Fig. 2), indicating that, as expected, Mg was discarded from both BChl *a*_*p*_ and BChlide *a*, and replaced by one or two protons.

BPhe *a*_*p*_, obtained from demetalation of the reference BChl *a*_*p*_, was used as a reference for MS and MS/MS mass spectrometry.

#### Demetalation of the particle sample extracts

In the MS-spectra of native particle samples obtained from cultures of *Rba. capsulatus*, the intensity of the signal at m/z 911.5/910.5, identified as BChl *a*_*p*_ according to its mass value obtained in the MS-spectra, was extremely low (Fig. 3 and Table 1). This signal also showed extremely low intensities in the MS-spectra of both particle sample extracts before and after acid treatment (Additional file 7), and demetalation efficiency could not be determined for this signal.

The MS-spectra of extracts from the particle samples obtained from cultures of *R. rubrum* showed an MS-signal of high intensity at m/z 882.6 compared to that obtained for native particles (compare Fig. 3a and Additional file 6A). As the BChl *a* produced by *R. rubrum*, in contrast to most natural Chl’s and BChl’s, contains the geranylgeraniol instead of the phytol isoprenoid ester [52–54], this signal was attributed to BPhe *a*_*g*_ the demetalation product of BChl *a*_*g*_ (see Table 2). The high intensity of this signal in the MS-spectrum of the particle sample extracts was considered to result from both demetalation of BChl *a*_*g*_ during the extraction process and to a more efficient detection of this compound in particle extracts than in native particle samples. Furthermore the MS-signal at m/z 905.5/904.5 was of low intensity in both particle sample extracts, before and after treatment with conc. acetic acid (Additional file 6). Consequently, demetalation efficiency was difficult to quantify for this compound.

The signal at m/z 632.2 present in the MS-spectra of extracts from particle samples isolated from cultures of *R. rubrum* decreased to 4.5% of its initial intensity, and was replaced by a signal at m/z 610.3 (Table 2 and Additional file 6). The loss of 22 m. u. from this signal after acid treatment well agreed with the loss of Mg which was replaced by two protons, as observed for the reference BChlide *a*. Thus, the m/z value of this signal together with the results of demetalation allowed to attribute it to BChlide *a*, and its demetalation product to BPheide *a* (see Fig. 2).

The intensity of the MS-signals at m/z 595.3 and m/z 616.2 only slightly decreased by treatment with conc. acetic acid (by 8.4 and 6.5%, respectively) (Table 2 and Additional file 7), indicating that these molecules did not contain Mg. In addition, the intensity of these signals did neither decrease after additional treatment of the sample spot with conc. HCl saturated with FeSO _4_, confirming that these molecules did not contain any metal.

### Tandem MS-spectra

#### Tandem MS-spectra of the references BChl *a*_*p*_ and BPhe *a*_*p*_

Consistent with literature reports [48, 55–57] the reference BChl *a*_*p*_ yielded a major product ion at m/z 632.2 corresponding to the loss of the phytyl moiety (278.3 m. u.) and representing BChlide *a* (see Fig. 2). The intensity of signals corresponding to the loss of additional fragments was less than 4% of that of the major ion (Table 3).

The reference BPhe *a*_*p*_ yielded a major product ion at m/z 610.3 also corresponding to the loss of the phytyl moiety and representing BPheide *a* (see Fig. 2). In this spectrum, however, additional signals were present, which derived from losses of a [-COOCH _3_] or a [-CH _2_-COOH] fragment (59 ±1 m. u.), of a [-CH _2_-CH _2_-COOH] fragment (73 m. u.) or of both fragments, yielding product ions at m/z 550.3 (±1 m. u.), m/z 537.3, and m/z 477.3, respectively (Table 3).

#### Tandem MS-spectra of the MS-signals of particle samples attributed to BChl *a*_g_ and BPhe *a*_g_

The low intensity signals at m/z 905.5/904.5 present in the MS-spectrum of the native particles samples isolated from the culture medium of *R. rubrum* (Fig. 3 and Table 1) yielded a major product ion at m/z 632.2 corresponding to the loss of a fragment of 273.3 or 272.3 m. u. The mass of this fragment well corresponded to that of the geranylgeranyl moiety (Fig. 2, Table 3) and the molecular ion, which showed the same m/z value as that obtained in the fragmentation of the reference BChl *a*_p_ (m/z 632.2), was assigned to BChlide *a*. Thus, the results of tandem MS-analysis confirmed the results of demetalation i.e. the presence of BChl *a*_g_ in the Se^0^-nanoparticle samples isolated from the culture medium of *R. rubrum.* Furthermore, this precursor ion yielded additional signals at m/z 572.3 and m/z 499.3, i.e. signals which were shifted by 22 m. u. compared to the second and the fourth signals obtained for the reference BPhe *a*_p_ (Table 3), thus indicating the presence of Mg in each of these product ions. This result once more confirmed the identification of the precursor ion at m/z 905.5/904.5 as BChl *a*_g_.

The signal at m/z 888.6, obtained in the MS-spectra of extracts of particle samples isolated from cultures of both bacterial species (Fig. 3 and Table 1) yielded the same fragmentation pattern in tandem MS as the reference BPhe *a*_p_ (Table 3). This result confirmed the presence of BPhe *a*_p_ in Se^0^-nanoparticle samples obtained from cultures of both *R. rubrum* and *Rba. capsulatus*. It was consistent with the fact that *R. rubrum* has been shown to contain BPhe *a*_p_ together with BChl *a*_g_ in its reaction center [52].

Fragmentation of the signal at m/z 882.6, obtained in the MS-spectra of both particle samples and particle sample extracts from cultures of *R. rubrum*, yielded a major ion at m/z 610.3 as in the fragmentation of the reference BPhe *a*_p_ (Table 3). In these cases, however, the fragment loss was 272.3 m. u. instead of 278.3 m. u., indicating that a geranylgeranyl moiety instead of a phytyl moiety was lost from this precursor ion (Fig. 2). This result was once more consistent with the presence of BChl *a*_g_ in the particle samples isolated from culture of *R. rubrum*, which was demetalated in the cells (see below) and under the acid condition used in the preparation of the samples, thus yielding BPhe *a*_g_. Furthermore, similarly to the fragmentation pattern obtained in the tandem MS of the reference BPhe *a*_p_, additional signals at m/z 550 (± 1 m. u.), m/z 537.3, and m/z 477.3, were present in the tandem MS of the MS-signal at m/z 882.6 (Table 3), thus confirming its identification as BPhe *a*_g_. Also in this case, the result of tandem MS-analysis was consistent with that of demetalation (see Table 2).

The ratio of phytol to geranylgeraniol alcohol component of the photosynthetic unit of *R. rubrum* grown anaerobically in the light was reported to be approximately 1:20 [54]. This ratio is consistent with a photosynthetic system composed of a reaction center surrounded by a ring of 16 *αβ* light harvesting heterodimers [58]. In our work we measured a mean ratio of 1:2.5 for the ratio of BPhe *a*_g_ to BPhe *a*_p_ (3 measurements, see Table 1). This discrepancy is likely the result of oxidative stress-induced demetalation of BChl *a*_g_ [59–61] in cultures of *R. rubrum* exposed to a toxic concentration of selenite, accompanied by the activity of a variant of the geranylgeranyl reductase (BchP) which is present in this bacterial species, and was reported to reduce BPhe *a*_g_, but not BChl *a*_g_ [62].

#### Tandem MS-spectra of the MS-signals of particle samples identified as porphyrins or bacteriochlorins according to their mass value

PPIX and PPIX-di-O-CH _3_, as well as the tandem MS-signal of PPIX-di-O-CH _3_ at m/z 577.3 and representing PPIX-O-CH _3_ were used as references.

Product ions obtained in the tandem MS of the reference PPIX-di-O-CH _3_ accounted for the losses of fragments of 59, 73, and 87 m. u. These fragments were attributed to the [-COOCH _3_], the [-CH _2_-COOCH _3_], and the [-CH _2_-CH _2_-COOCH _3_] group, respectively, which were lost from the propionyl side chains of the tetrapyrrole ring (Additional file 8). This result was in good agreement with literature reports indicating that losses of C13 and C17 substituents of porphyrin molecules yield the most intense signals in MS/MS, while signals corresponding to the loss of other substituents are of very low intensity [55]. A similar fragmentation pattern was obtained for the reference PPIX, but with the difference that the propionic acid side chains were not esterified, thus yielding fragment losses of 45, 59 and 73 m. u. corresponding to the [-COOH], the [-CH _2_-COOH], and the [-CH _2_-CH _2_-COOH] group, respectively (Fig. 4).

Fragments containing one supplementary mass unit compared to those indicated above, (46, 60, 74, and 88 m. u.) were considered to result from the transfer of a hydrogen atom from the product ion to the fragment, accompanied by a McLafferty rearrangement in the product ion [63].

The most intense signals obtained were derived from the cleavage of the *β* bond of the C13- or C17-propionyl moiety. Cleavage of the *α* or *γ* bond showed significantly less intense signals (Figs. 4, 5 and Additional file 8). These results were consistent with those obtained using Electron Ionisation Mass Spectrometry [64] and Electrospray Ionisation-MS/MS [65] analyses of porphyrins.

Comparison of the tandem MS-spectra obtained for the MS-signals of the Se^0^-nanoparticle samples at m/z 563.3 and m/z 577.3 to those of the references PPIX and PPIX-O-CH _3_, respectively, showed that fragment losses and masses of product ions of the respective analyte and reference were identical on the whole fragmentation pattern (Figs. 4 and 5), despite differences in signal intensities between reference molecules and respective signals obtained with Se^0^-nanoparticle samples. These modifications of signal intensities were assumed to result from differences in the conformation of the respective molecules but were difficult to explain. We considered, however, tandem MS-analysis to confirm the provisional identification of the MS-signals at m/z 563.3 and m/z 577.3, obtained in the MS-spectra of the Se^0^-nanoparticle samples, as PPIX and PPIX-O-CH _3_, respectively (Table 1).

The MS-signal at m/z 557.1 obtained in the MS-spectra of Se^0^-nanoparticle samples isolated from cultures of both bacterial species (Table 1) showed an identical m/z value as the major product ion of the MS-signal at m/z 616.2, and was attributed to the pyro-derivative of the corresponding molecule (see below).

Signals with m/z values below 550 (i.e. m/z 537.2, m/z 517.2, m/z 504.2, m/z 499.2, m/z 477.2) obtained in the MS-spectra of Se^0^-nanoparticle samples isolated from cultures of either bacterial species (Table 1) were found to be identical to those of product ions of BChl *a*_g_, BPhe *a* (BPhe *a*_p_ and BPhe *a*_g_), BPheide *a*, PPIX-O-CH _3_, and PPIX. Thus, they were considered to represent degradation products of BChl *a* or of BChl *a* biosynthesis intermediary metabolites (Table 3, Figs. 4 and 5).

The signal for pyro-BPheide *a* (m/z 550.3) was hardly detected in the MS-spectra of the Se^0^-nanoparticle samples obtained from cultures of either *R. rubrum* and *Rba. capsulatus* despite the fact that this compound was reported to accumulate to significant amounts in aged cultures of *Rba. sphaeroides* [21] and that pyro-Pheide *a* likewise accumulated in stationary phase cultures of *Chlamydomonas reinhardtii* transferred to anaerobiosis at the end of the growth phase [25]. The very low intensity of the signal for pyro-BPheide *a* in our particle samples was attributed to the fact that the particles were harvested at early stationary phase (see “Methods” section).

#### Tandem MS-analysis of the MS-signals at m/z 599.2/598.2

Comparison of the tandem MS-spectrum of the MS-signal at m/z 599.2/598.2 with that of the reference PPIX-O-CH _3_ (m/z 577.3) showed that an identical fragmentation pattern was obtained, which was shifted by 21 to 22 m. u. in the analyte compared to the reference, indicating that the structure of these molecules were very similar (Fig. 6, and Table 4). This mass difference well agreed with the replacement of three or two protons by Mg in PPIX-O-CH _3_, allowing the assignment of the molecule yielding the MS-signal at m/z 599.2/598.2 to Mg-PPIX-O-CH _3_.

#### Tandem MS-analysis of the MS-signal at m/z 595.3

Fragmentation of this MS-signal (Fig. 7) did not yield any molecular ion corresponding to the cleavage of an acetoxy group ([-CO-CH _3_], 43 m. u., *ε* cleavage), typical for the fragmentation of BPheide *a* (see Table 5 and Fig. 8). This indicated m/z 595.3 to represent an intermediary metabolite of BChl biosynthesis containing a C3-vinyl group instead of a C3-acetoxy group. As, in addition, this compound was metal-free (Table 2 and Additional file 7), we assigned it to the dephytylated and demetalated form of one of the intermediates upstream of 3-vinyl-bacteriochlorophyllide a hydroxylase, which catalyses the first step in the formation of the C3-acetoxy group of BChl *a* [66]. The most intense MS/MS-signal was for the loss of a 74 m. u. fragment. According to the fragmentation patterns obtained for the references PPIX and PPIX-O-CH _3_, this fragment was attributed to the cleavage of a *β* bond, indicating the presence of a [-CH _2_-CH _2_-COOCH _3_] side chain in the precursor ion. This assignment was consistent with the presence of a signal of relatively low intensity corresponding to the loss of a 88 m. u. fragment (*α*_1_ cleavage). A further signal corresponding to the loss of 104 m. u. was attributed to two simultaneous *β* cleavages (see above), i.e. the concomitant losses of a 73 m. u. fragment ([-CH _2_-COOCH _3_]) and of a 31 m. u. fragment. This latter fragment was assumed to derive from the *β* cleavage of a [-CH _2_-CH _2_-OH] side chain likely present in the precursor ion (Fig. 7b). Consistent with this proposition a signal of low intensity, corresponding to the *α* cleavage of the [-CH _2_-CH _2_-OH] side chain (45 m. u., *α*_2_ cleavage) or to the *α* cleavage of the [-COOH] group (45 m. u., *α*_3_ cleavage) was also present in this tandem MS. The possible presence of a [-COOH] group in the analyte, together with the relatively high intensity of the signal obtained at m/z 491.3 and corresponding to the loss of 104 m. u. (two fragments) led to the assumption that the MS-signal at m/z 595.3 could represent a degradation product of a demetalated protochlorophyllide (PChlide) with substituents bound not only to the C13 and C17 carbon atoms, but also to the C15 carbon atom of the tetrapyrrole ring, hence containing an opened isocyclic ring. Indeed, the intensity of the fragment at m/z 491.3 was consistent with a large number of double fragment losses representing a total of 104 m. u. (Fig. 7a). Also consistent with the structure proposed in Fig. 7b was the large number and low intensity of the signals proposed to represent the loss of three fragments. Ring opening could have resulted from the oxidative conditions prevailing when selenite was reduced in the presence of glutathione (see Discussion). The proposed structure corresponds to the methyl ester of a demetalated PChlide with opened isocyclic ring.

According to the total number of carbon atoms expected to be present in the substituents of the C13 and C15 carbon atoms of the tetrapyrrole molecule after opening of the isocyclic ring, one [-COOH] group and one [-CH _2_-CH _2_OH] group were considered to represent the 45 m. u. fragment.

Consistent with the proposed porphyrin structure, this molecule yielded an MS-signal with odd m/z value, i.e. corresponding to a protonated molecular ion, as obtained for the references PPIX and PPIX-O-CH _3_. The mean molecular mass of the protonated molecular ion measured on the MS-spectra yielded 595.302 ± 0.012 m. u. (4 measurements). It well agreed with the theoretical mass of 595.284 (*M*= C _35_*H*_39_*N*_4_*O*_5_) calculated using a monoisotopic composition of the molecule, thus confirming the elemental composition proposed for this compound.

However, as no reference was available for this compound, its chemical structure remains to be confirmed by further analyses.

#### Tandem MS-analysis of the MS-signal at m/z 616.2

According to demetalation experiments the molecule yielding the MS-signal at m/z 616.2, and representing the most intense signal obtained in the MS-spectrum of the Se^0^-nanoparticle samples isolated from cultures of *Rba. capsulatus* (Fig. 3), was also metal-free (Table 2 and Additional file 7). According to its fragmentation pattern its chemical structure was similar to that of the reference BPheide *a*. Indeed, most of the signals obtained in the tandem MS-spectra of BPheide *a* (m/z 610.3) were also present in the tandem MS-specta of the MS-signal at m/z 616.2 but shifted by 6-7 m. u. (Fig. 8 and Table 5). This included the most intense signal with a loss of 59 m. u., which was attributed to the loss of a [-CH _2_-COOH]- or a [-COOCH _3_]-group. It was consistent with the cleavage of the *β* bond of the C17-propionyl side chain of BPheide a [-CH _2_-COOH] or of its *δ* bond [-COOCH _3_]. A signal was also common for the loss of a 73 m. u. fragment which was attributed to the cleavage of the *α* bond of the C17 side chain. Signals representing the concomitant losses of two neutral fragments with total masses of 89, 105, 118, 133 and 148 m. u. were also obtained in the tandem MS-spectra of both the reference BPheide *a* and the molecule yielding the MS-signal at m/z 616.2 (Table 5), thus confirming the structure similarity between these compounds.

The most important differences between these spectra concerned signal intensities. The tandem MS of m/z 616.2 yielded significantly lower singal intensity for most product ions when compared to that of the corresponding product ions obtained for the reference BPheide *a*. Particularly large differences of intensity were obtained for signals representing a total loss of more than 148 m. u.. These signals were nearly absent from the tandem MS spectrum of m/z 616.2, while their intensity was comprised between 5 and 10% of the base peak in the spectrum of the reference BPheide *a* (Fig. 8a and Table 5).

Low signal intensities were assumed to result from the extension of the bacteriochlorin *π*-resonance system to the C7-O and C18-O bonds of this molecule together with differences in the conformation of the molecules associated with the particles compared to reference molecules, as it was also proposed for PPIX and PPIX-O-CH _3_. The proposed structure (Fig. 8b) is consistent with the most probable hydroxylation and demetalation of 3-acetoxy chlorophyllide *a*, the substrate of COR, which show high structure similarity with BPheide *a*. Degradation of this metabolite is proposed to take place at the active site of the chlorin reductase (COR) in the presence of the ROS produced during selenite reduction (see “Discussion” section). Selection of the hydroxylation sites was based on the relatively high reactivity of the carbon atoms which are not part of the aromatic delocalization system [67].

Also consistent with the bacteriochlorin structure proposed for the molecule represented by the MS-signal at m/z 616.2 was its even m/z value, which corresponded to a molecular ion radical as obtained for the reference BPheide *a*.

Its exact mass as determined by MALDI-TOF mass spectrometry yielded 616.212 ± 0.005 m. u. (5 measurements). It closely agreed with a theoretical exact mass of 616.217 (*M*= C _32_*H*_32_*N*_4_*O*_9_) calculated for the monoisotopic composition of the compound proposed in (Fig. 8b). The low fractional part of this mass is consistent with the relatively large number of oxygen atoms it contains as compared to BPheide *a* (*M*= (C _35_*H*_38_*N*_4_*O*_6_), which gives a theoretical mass of 610.279 m. u. On the other hand the low difference between the measured and the theoretical mass we obtained confirmed the elemental composition we proposed for this compound.

However, as in the case of m/z 595.3, the structure of m/z 616.2 has to be confirmed by further analyses.

In addition to signals representing BChl *a*_p_, BPhe *a*_p_, BPheide *a* and intermediary metabolites of the BChl *a* biosynthesis pathway, the MS-spectra of the Se^0^-nanoparticule samples isolated from cultures of *Rba. capsulatus* showed signals at m/z 786.6, m/z 808.6 and m/z 824.6 (Fig. 3). Tandem-MS analysis of these signals led to the conclusion that they represented a phosphatidylcholine lipid (Additional file 5).

Altogether these results indicated that not only BChl *a*, BPhe *a* and BPheide *a* accumulated in cells grown in the presence of selenite, as reported for stationary phase cultures of phototrophic organisms grown in the absence of selenite [21, 25], but that intermediary metabolites of the BChl biosynthesis, as PPIX, PPIX-O-CH _3_, BChlide *a* and, most likely, Mg-PPIX-O-CH _3_ (Figs. 4, 5, and 6) as well as the proposed oxidation products of intermediary metabolites of this pathway (m/z 595.3 and m/z 616.2) (Figs. 7 and 8) also accumulated in cells of *R. rubrum* and *Rba. capsulatus* grown in the presence of a toxic concentration of selenite. Interestingly, these metabolites were associated with the Se^0^-nanoparticles these bacteria expelled in the last step of selenite detoxification.

## Discussion

### Particle size

The diameter of bacterially-produced Se^0^-nanoparticle reported in the literature varies largely depending on the bacteria considered, and, most likely, on other parameters such as growth and measurement condition (see below). The smallest reported diameter of 20 nm was measured for particles produced by *Veillonella atypica* [11]. Significantly larger diameters of nearly 400 nm were reported for particles produced by *Pseudomonas* sp strain CA5 [68], and *Bacillus selenitireducens* [69]. But, in most cases the reported particle diameter varied between 100 nm and 200 nm [8, 9, 70, 71]. The Se^0^-nanoparticle diameter reported in the present work shows a large dispersion, with ratios of up to 1:9 between the smallest and the largest particles. We propose that the largest particles resulted from coalescence of the smallest ones. Indeed, coalescence of uncoated as well as of surfactant-coated gold nanoparticles dispersed in aqueous solution was observed using dynamic in situ TEM imaging [72]. These authors showed that pairs of nanoparticles underwent a rapid approach when they were separated by a minimal distance which depended on the surface-bound molecules. The pairwise approach resulting from particle movements due to thermal fluctuations was followed by a sudden jump to contact and pairwise attachment. We therefore assume that the Se^0^-nanoparticles formed in the cells of *R. rubrum* and *Rba. capsulatus* during selenite reduction most likely displayed, initially, very small diameters, and that larger particles resulted from coalescence of the smallest ones. This proposition is consistent with observations that particle diameters increased with the time elapsed between their formation and measurement. For example, particles produced by *Bacillus mycoides* showed a diameter between 50 nm and 100 nm when measured 6 h after the beginning of selenite reduction, and between 50 and 400 nm 42 h later [10]. Increase of particle diameter with time has also been reported by Garbisu et al. [73].

The relatively small particle diameters obtained in this work, compared with those obtained in most cited reports, were likely the result of early harvesting of the particles, i.e. 2 days after the end of selenite reduction, (see “Methods” section).

### Association of BChl *a*, BPhe *a* and BPheide *a* with Se^0^-nanoparticle samples

According to the results of MALDI-TOF analyses reported here, *R. rubrum* and *Rba. capsulatus* grown in the presence of a toxic concentration of selenite (0.5 mM) accumulated BChl *a*, BPhe *a* and BPheide *a* (Fig. 3 and Tables 1, 2 and 3), which were shown to accumulate in stationary phase cultures of *Rba. sphaeroides* grown in absence of selenite [21]. Accumulation of these metabolites in particle samples was therefore not attributed to selenite stress.

Concerning BChl accumulation we observed that MS-signals for BChl *a*_p_ and BChl *a*_g_ in the MS-spectra of the native particle samples obtained from cultures of both *R. rubrum* and *Rba. capsulatus* were very low, (Table 1 and Fig. 3), while BChl *a*_p_ represented the predominant accumulated metabolite in early stationary phase cultures of *Rhodobacter sphaeroides* grown in the absence of selenite (Haidl et al., 1985). This discrepancy suggested that BChl’s were possibly not efficiently detected in our samples. Inefficient detection of BChl’s in the native particle samples is consistent with the large increase of signal intensity for BPhe *a*_p_ and BPhe *a*_g_ in the MS-spectra of the particle sample extracts, particularly in the demetalated particle sample extracts (Additional files 6 and 7), compared to the signal intensity of the respective precursors, BChl *a*_p_ and BChl *a*_g_, obtained in native particle samples. Low detection efficiency of BChl’s in native particle samples was attributed to interactions of these compounds with other components of the particles.

Interestingly, BPheide *a* was shown to stay associated with the particles after the detergent-washing procedure, that is after solubilization of MVs (Additional file 4). We therefore considered that this compound was tightly bound to the Se^0^-nanoparticles the bacteria expelled in the last step of selenite detoxification.

### Association of intermediary metabolites of BChl *a* biosynthesis with Se^0^-nanoparticle samples

As already mentioned, cultures of *R. rubrum* and *Rba. capsulatus* exposed to a toxic concentration of selenite not only accumulated BChl *a*, BPhe *a* and BPheide *a*. The Se^0^-nanoparticle samples were associated, additionally, with intermediary metabolites of the BChl biosynthesis, i.e. PPIX, PPIX-O-CH _3_ (Figs. 4 and 5), Mg-PPIX-O-CH _3_ (Fig. 6) and BChlide *a* (Table 2), as well as with compounds which, according to MS and tandem MS measurements complemented with demetalation experiments, corresponded to oxidation products of the demetalated protochlorophyllide methyl ester and of the demetalated 3-acetoxy chlorophyllide *a*, the demetalated substrates of DPOR and COR, respectively (Figs. 7 and 8).

As in the case of BPheide *a* all these compounds remained associated with the Se^0^-nanoparticles after the detergent-washing procedure (Additional file 4), indicating that they were tightly bound to the particles and not, or not only, with MVs which contaminated the particle samples.

Accumulation of these compounds in cells was attributed to inhibition of enzymes of the BChl biosynthesis pathway resulting from the formation of molecular oxygen and ROS produced during the reduction of selenite. Indeed, as indicated in the introduction, purple phototrophic bacteria were shown to contain large amounts of glutathione [26, 27], and reduction of selenite to occur intracellularly [12, 13]. Considering the particularly high reactivity of selenite with reduced glutathione measured in the in vitro reactions [30], which led to the formation of superoxide anions, we considered that this reaction most likely also takes place in cells of *R. rubrum* and *Rba. capsulatus* exposed to selenite. Consequently, the toxic effects of selenite on the metabolism of these bacteria may largely result from the formation of superoxide anions as well as from its degradation products; i.e. hydrogen peroxide and molecular oxygen, which are liberated during reactions catalyzed by SOD(s) and catalase(s), respectively. It is known that superoxide anions, hydrogen peroxide and molecular oxygen affect gene expression and/or enzyme activities [6, 35, 37, 38, 74, 75]. Oxygen-mediated downregulation of gene transcription has been reported for several genes of the BChl biosynthesis pathway in *Rba. capsulatus*, thus leading to accumulation and excretion of intermediary metabolites of this pathway [76]. We could therefore propose that accumulation and excretion of intermediary metabolites of BChl biosynthesis in cultures of *R. rubrum* and *Rba. capsulatus* grown in the presence of a toxic concentration of selenite, as shown here, resulted, at least in part, from downregulation of gene expression.

However, transcription of the gene encoding Mg-PPIX-O-CH_3_ oxidative cyclase (bchE), was shown not to be regulated by oxygen [77]. Though, despite of this result, Mg-PPIX-O-CH_3_, the substrate of BchE (m/z 599.2), accumulated in cultures of both *R. rubrum* and *Rba. capsulatus* grown in the presence of 0.5 mM selenite (Figs. 3, 6 and Table 4). We therefore propose that accumulation of this metabolite resulted from inhibition of the catalytic activity of Mg-PPIX-O-CH_3_ oxidative cyclase and not from inhibition of gene transcription.

As indicated in Table 6, five enzymes of the Mg-branch of the BChl biosynthesis pathway leading to BChl *a*, are potential targets for inactivation in the presence of molecular oxygen and ROS: (i) magnesium chelatase, containing a highly oxygen sensitive [4Fe-4S] cluster present in its BchH subunit [78]. Inhibition of this enzyme may explain accumulation of its substrate PPIX (m/z 563.3) in the cultures and association of PPIX with the Se^0^-nanoparticles (Fig. 3 and Table 1); (ii) magnesium-PPIX methyltransferase (BchM), which uses the oxygen-sensitive S-adenosyl-methionine (SAM) as a methyl group donor [79] and is activated and stabilized by BchH, the oxygen-sensitive substrate-binding subunit of Mg-chelatase [80, 81]. Although we did not detect Mg-PPIX, the substrate of BchM, in any Se^0^-nanoparticle sample investigated, we consider that similarly to Mg-PPIX-O-CH_3_ (see below), this molecule was demetalated in the cells and/or during the particle isolation process, thus contributing to the presence of PPIX (m/z 563.3) in the MS-spectra of the Se^0^-nanoparticle samples (Fig 3 and Table 1); (iii) BchE, which also contains a highly oxygen-sensitive [4Fe-4S] cluster in its active site [77]. Inactivation of this enzyme would lead to accumulation of its substrate, Mg-PPIX-O-CH_3_. Thus, consistent with tandem MS analysis, the MS-signal at m/z 599.2/598.2 (Fig. 6 and Table 4) most likely represented Mg-PPIX-O-CH_3_. This molecule has been reported to be unstable in light and to lose Mg at a pH below 7.0 [82], thus explaining the relatively low intensity of this signal and, consequently, the high intensity of the signal for PPIX-O-CH_3_(m/z 577.3) in the MS-spectra of the Se^0^-nanoparticle samples isolated from cultures of both *R. rubrum* and *Rba. capsulatus*(Fig. 3 and Table 1).

**Table 6:** Enzymes of the BChl *a* biosynthesis pathway that are particularly sensitive to oxygen

Gen name	Enzyme name	Functional groups sensitive to oxygen
bchI bchD bchH	Mg-chelatase	BchH contains the particular **cystein motif CX**_**2**_**CX**_**3**_**CX**_**14**_**C**, which coordinates a **[4Fe - 4S] - cluster**. This iron - sulfur cluster is particularly sensitive to oxygen and is transformed to a **[3Fe - 4S] cluster** in its presence. [78].
bchM	Mg-PPIX-O-methyltransferase	The methyl group is supplied by **S - adenosyl - methionine**. The oxygen - sensitive BchH has been proposed to be the substrate - binding protein of BchM [79, 80]
bchE	Mg - PPIX - O - CH_3_ oxidative cyclase	BchE contains a **conserved CX**_**3**_**CX**_**2**_**C cystein motif**, which coordinates an oxygen - sensitive **[4Fe - 4S] - cluster** [77].
bchL bchN bchB	Dark allowed protochlorophyllide oxidoreductase (DPOR)	The reductase component BchL contains two **conserved cysteine residues** and the BchL- dimer coordinates an oxygen - sensitive **[4Fe - 4S]-cluster** [89]. The catalytic component (BchN-BchB)_2_ also contains conserved cysteines, which coordinate [4Fe - 4S]-clusters, however these are not oxygen-sensitive [129]
bchX bchY bchZ	Chlorin reductase (COR)	BchX, BchY and BchZ are enzyme subunits structurally and functionally similar to BchL, BchN, and BchB, respectively [90, 91]. It has been shown that the subunit dimers (BchL)_2_ and (BchX)_2_ of DPOR and COR, respectively, which drive electrons to the substrates of these enzymes, are exposed to the cytoplasm [91, 92], i.e. to selenite and to the ROS produced during selenite reduction.

Note that each of these enzymes contain SAM, and/or [4Fe-4S] clusters coordinated by cysteine residues, that are potential targets for inactivation by both selenite and the ROS produced during the reduction of selenite to elemental selenium. These potential targets are highlighted using boldface characters

Consistent with the proposed inhibitory effect of selenite toxicity on BChl biosynthesis, is the large induction of cysteine desulfurase (IscS or iron sulfur cluster S protein), an enzyme involved in [Fe-S]-clusters repair, in cultures of *E. coli* grown in the presence of a toxic concentration of selenite [32, 83].

The [4Fe-4S] clusters of these first three oxygen-sensitive enzymes of the Mg-branch of the BChl *a* biosynthetic pathway were reported to be shielded from the cytoplasm [84–86]. Consequently, their active sites are assumed not to be directly inactivated by the ROS produced during selenite reduction. They were most likely inactivated by molecular oxygen [37] produced during the degradation of O_2_^·^^−^ and H_2_O_2_ by SOD’s and catalases, respectively. Indeed, molecular oxygen is small enough to penetrate all but the most shielded active sites of redox enzymes [87], while the polarity of H_2_O_2_ and the negative charge of O_2_^·^^−^, prevent these molecules reaching active sites which are shielded from the cytoplasm.

The fourth and fifth oxygen-sensitive enzymes of the Mg-branch of the bacteriochlorophyll biosynthesis pathway are the dark allowed (or dark operative) protochlorophyllide oxidoreductase (DPOR) and chlorin reductase (COR), which not only share significant amino acid sequence identity and structure similarity with each other, but also with nitrogenases [50, 88–91]. By contrast to the [4Fe-4S] clusters present in the first three oxygen-sensitive enzymes of this pathway, the [4Fe-4S] clusters contained in the subunit dimers (BchL)_2_ and (BchX)_2_ of DPOR and COR, respectively (see Table 6), which drive electrons to the substrates of these enzymes, were shown to be exposed to the cytoplasm [91, 92], i.e. to selenite and to the ROS produced during selenite reduction. Consequently, the Fenton reaction, producing highly reactive OH radicals (OH^·^) from H_2_O_2_ in the presence of Fe^2+^ [87, 93, 94] may take place with high probability in DPOR and COR, leading to oxidation of the respective substrate of these enzymes.

Furthermore, ferredoxin(s), which contain(s) water-exposed [Fe-S] clusters [95, 96] are proposed to function as electron donor(s) to DPOR and COR [97, 98]. Consequently, they represent a further target for OH^·^ formation in the vicinity of the active sites of DPOR and COR.

Due to the particularly high reactivity and, thus, short life time and small radius of action of OH^·^ [99], oxidation products are expected only at, or very close to the active sites of DPOR and COR. Consequently, only the substrates or reaction products of these enzymes could be exposed to OH^·^, thus explaining that no other oxidized substrates or reaction products of the BChl biosynthesis pathway were detected in our experiments.

As the MS-signal at m/z 595.3 was shown to represent a metal-free compound (Table 2), its tandem-MS analysis led to the proposition that it should represent the methyl ester of an oxidation product of demetalated PChlide (Fig. 7b), i.e. the methyl ester of a demetalated and oxidized product of the substrate of DPOR with opened isocyclic ring. This proposition is inline with two different reports ([100] and chapter 5 of [101]), indicating that nucleophilic attack on the C13^1^-carbon atom of this ring cleaves the C13^1^-C13^2^ bond, thus leading to the formation of a carboxylamide group. We propose that a similar reaction occurs with OH^·^ produced by the Fenton reaction taking place in (BchL)_2_, the cytoplasm-exposed electron transporter of DPOR. This reaction would lead to the formation of a [-COOH] group bound to the C13-carbon atom and a [-CH_2_-COOCH_3_] group bound to the C15-carbon atom of the tetrapyrrole ring. Further reactions leading to the structure proposed in Fig. 7b can only be speculative, possibly resulting from a second nucleophilic attack followed by reduction at the expense of the cysteine residues which coordinate the [4Fe-4S] cluster of the active site of DPOR or by ferredoxin, which is proposed to function as electron donor to DPOR (see above).

The suggestion that the MS-signal at m/z 595.3 represents a degradation product of PChlide is supported by the fact that no other MS-signal corresponding to a PChlide degradation product could be identified in particle samples or in particle sample extracts. Absence of such a compound would be surprising in *Rba. capsulatus*, which does not possess a very efficient peroxidase system (see below), and, consequently, whose DPOR active site may be exposed to hydrogen peroxide and produce OH^·^. Methylation of this degradation compound can possibly be explained by the activity of a radical SAM protein. This protein is reported to catalyze diverse reactions, including unusual methylations [102].

The MS-signal at m/z 616.2 was also shown to represent a metal-free compound (Table 2). Consequently, it could neither be identified as 3-vinyl-BChlide (m/z 616.1, Table 1) [50], nor as protoheme [51] as provisionally expected according to the mass of this compound obtained in the MS-spectra (see Table 1). Consistent with the absence of an MS-signal for protoheme in our samples, it has been reported that BChl *a* is synthesized in nearly 100 fold excess over heme in *Rba. sphaeroides* grown under anaerobic phototrophic condition [103]. Protoheme was therefore unlikely to be detected in our experiments.

Tandem MS analysis of m/z 616.2 led to the proposition that it represented an oxidized derivative of the demetalated 3-acetoxy chlorophyllide *a*, the demetalated substrate of COR (Fig. 8b). Similarly to the reaction proposed to lead to the formation of m/z 595.3 (see above), oxidation of 3-acetoxy chlorophyllide *a* was assumed to be performed by OH^·^ formed in (BchX)_2_, the cytoplasm-exposed electron transporter of COR [91, 92]. As the tandem MS-spectrum of this MS-signal showed high similarities with that of BPheide *a*, the demetalated reaction product of COR, the structure of the corresponding compound was considered to be similar to that of the demetalated 3-acetoxy chlorophyllide *a*, the substrate of COR, the structure of which is very similar to that of BPheide *a*. Consistent with the proposed structure, OH^·^ was shown to replace H atoms in both aliphatic and aromatic compounds, leading to the formation of hydroxylated reaction products [104, 105]. As already mentioned, selection of the hydroxylation sites was based on the relatively high reactivity of the carbon atoms which are not part of the aromatic delocalization pathway [67].

The difference between the chemical structure of m/z 595.3 and that of m/z 616.2 is striking. It likely results from specific amino acid composition and three dimensional conformation of the respective active sites of DPOR and COR [91, 106], leading to different sites of OH^·^ nucleophilic attack on the substrate of the respective enzymes.

The Se^0^-nanoparticle samples from both bacterial species were associated, additionally, with BChlide *a*, the substrate of BChl synthase. This compound was identified according to its mass (Table 1, Fig. 3) as well as to results of demetalation (Table 2). Similarly to the other signals discussed above, accumulation of BChlide in cultures of *R. rubrum* and *Rba. capsulatus* grown in the presence of a toxic concentration of selenite, was attributed to ROS-mediated enzyme inhibition of BChl synthase.

Consistent with this proposition, analysis of the structures of Chl synthase and BChl synthase using the transmembrane topology prediction server revealed that these enzymes are membrane proteins composed of nine membrane spanning helixes [107, 108]. Site-directed mutagenesis demonstrated that the geranylgeranyl-diphosphate- or phytyl-diphosphate-binding region is located in a loop between the second and the third membrane spanning helixes. Consequently, the geranylgeranyl-diphosphate- or phytyl-diphosphatee-binding site of these enzymes is exposed to the cytoplasm, i. e., to the ROS produced during the reduction of selenite with glutathione, which are known to damage protein structure [74]. Moreover, a cysteine residue has been identified in this substrate-binding site in the BChl synthase of both *Rba. capsulatus* and *Rba. sphaeroides*. The reactivity of selenite with –SH groups represents, therefore, a further clue for inactivation of BChl synthase in cells of these bacteria exposed to a toxic concentration of selenite and, consequently, accumulation of its substrate.

The presence of large amounts of the presumed oxidized derivatives of BChl metabolites (m/z 595.3 and m/z 616.2) in the Se^0^-nanoparticle samples isolated from cultures of *Rba. capsulatus* compared to the amount of such derivatives excreted by *R. rubrum*, (Fig. 3 and Table 1) was consistent with differences between the two bacterial species in the composition of enzymes involved in protecting the cells from oxidative stress. Indeed, *Rba. capsulatus* contains a single SOD that is homologous to Fe/Mn-SOD [109], while genes for both Fe/Mn- and Cu/Zn-SOD are present in the genome of *R. rubrum* (UniProt G2T9Q6 and Q2RTG3). Furthermore, one monofunctional catalase and one bifunctional catalase-peroxidase were identified in *Rba. capsulatus* [110], while three monofunctional catalases and two bifunctional catalase-peroxidases are known in *R. rubrum* [111]. Also consistent with the excretion by *Rba. capsulatus* of large amounts of compounds assumed to represent oxidation products of BChl biosynthesis intermediates is the relatively slow process of selenite reduction in this bacterial species. Indeed, *Rba. capsulatus* reduces selenite from the beginning to the end of the growth phase [19] causing these cells to be exposed to a large amount of ROS for the entire time period during which BChl is synthesized. By contrast, *R. rubrum* quickly detoxifies selenite at the end of the growth phase [12, 19], thus being exposed to the presence of ROS for a significantly shorter time, at which the rate of BChl biosynthesis is slowed down.

MS-signals representing C13^2^-OH derivatives (allomers) of bacteriochlorin molecules at m/z 647.3 or 648.3 (BChlide *a* allomer), at m/z 921.5 (BChl *a*_g_ allomer) and at m/z 926.5 (BChl *a*_p_ allomer) (Fig. 3), were of very low intensity in the particle samples, despite the oxidative condition prevailing in the cultures during selenite reduction (Fig. 3). This result can be explained by the kinetics of allomerization. This reaction is reported to involve, in a primary step, the oxidation of the enolate anion of the isocyclic ring by triplet oxygen [42, 112]. As (B)Chl is significantly more stable in its keto form than in its enol form [113, 114], allomerization is a slow process. Completion of the reaction in samples of Chl stirred in the presence of air or even in the presence of pure oxygen in the dark is only attained after 2.5 days or more [112, 115, 116]. In cultures of the purple phototrophic bacteria growing under anaerobic condition in the presence of 0.5 mM selenite, the concentration of molecular oxygen produced during the reduction of selenite remains lower than 0.5 mM over the whole reduction time, thus much lower than in cultures stirred in the presence of air or oxygen. Consequently, allomerization is expected to be a very slow process in these cultures, which explains the low intensity of the MS-signals for allomers.

#### General aspects of selenite toxicity

It seems clear that the ROS produced during the reduction of selenite with glutathione do not only affect BChl metabolism. Indeed, as reported in the literature, ROS potentially affect, more generally, the structure, composition and metabolism of DNA, RNA, proteins and lipids [74, 75]. For example, cultures of cells which are not particularly resistant towards selenite, such as mammalian cells, were shown to be blocked in gene transcription already in the presence of micromolar levels of selenite [6]. This deleterious metabolic effect is proposed to largely result from the high level of cellular glutathione and its strong reaction with selenite, leading to the production of ROS within the first minutes of exposure [30]. Consequently, culture growth of glutathione-containing organisms in the presence of millimolar levels of selenite, presupposes that gene transcription is largely preserved from damages caused by selenite, as well as by ROS produced during the reduction of selenite with glutathione (see Introduction). It infers that these organisms are able to control cellular accumulation of selenite. We propose that regulation may take place at the level of membrane transport of this oxyanion. This is consistent with the delay of selenite reduction to stationary phase observed in many species of proteobacteria [7, 12, 117], indicating that these organisms are able to control selenite uptake. It is also consistent with results reported by Sarret et al. [118]. Using X-ray absorption spectroscopy these authors investigated accumulation of selenite and its various transformation forms in cells of *Ralstonia metallidurans* (*β*-proteobacteria). They observed that selenite was slowly transported into the cells during many hours after addition to the bacterial cultures, and that uptake becames significantly faster after this adaptation period. We propose therefore that control of selenite uptake likely allows to limit its distribution to the periphery of the cytoplasm, thus decreasing its deleterious metabolic effects.

Involvement of glutathione in the reduction of selenite in purple phototrophic bacteria, accompanied by the formation of ROS as observed in the in vitro reaction [30], is consistent with the large induction of glutathione reductase as well as of SOD’s and catalase in cultures of *E. coli* grown in the presence of a toxic concentration of selenite [32]. It also well agrees with the fact that absence of glutathione in *E. coli* and *S. typhimurium* (*ΔgshA* mutants) relieved the hypersensitivity to selenite of strains devoid of SOD (*ΔsodA*, *ΔsodB* mutants) [32]. On the other hand, selenite stress was also accompanied by a large induction of the thioredoxin/thioredoxin reductase system [13, 32], which was shown to be involved not only in the degradation of selenodiglutathione [119] but also in the first step of the reaction [120]. Reduction of selenite by the thioredoxin/thioredoxin reductase enzymatic system may explain, at least in part, the significant selenite reduction activity of cells deprived of glutathione as observed previously [19]. Furthermore, any freely exposed -SH groups contained in small metabolites and macromolecules are expected to represent potential electron donors to selenite, thus explaining the large toxicity impact of this oxyanion on the metabolism of living cells when it succeeds entering the cytoplasm.

Further selenite toxicity effects can be expected to take place in the periplasm as well. Indeed, the phage shock protein A (PspA) was shown to be very strongly induced in *E. coli* grown in the presence of a toxic concentration of selenite [32], and the *psp* protein system to respond to extracytoplasmic stresses and to be involved in the maintenance of redox equilibrium in the periplasm as well as in membrane structure and function [121, 122]. Deleterious effects of selenite on the structure and function of periplasmic and membrane proteins may result from interactions of this oxyanion with the large number of thiol-disulfide reactions taking place in the periplasm [123, 124].

Tight regulation of the membrane transport of selenite together with induction of the synthesis of proteins protecting cells against oxidative stress may represent key responses to selenite stress in selenite-resistant organisms reducing selenite intracellularly.

### Complexity of the chemical composition of the particles

MS-spectra of native Se^0^-nanoparticle samples isolated from cultures of *Rba. capsulatus* and embedded in the DHB-matrix, not only showed signals representing BChl *a* as well as intermediary metabolites of BChl *a* biosynthesis and degradation. They also displayed signals for a phosphatidylcholine lipid (Additional file 5). As these lipids were reported to represent the most sensitively detectable phospholipids [125], we expect that other lipids, not formally identified in this work, may be associated with the Se^0^-nanoparticles produced by *R. rubrum* and *Rba. capsulatus*. It could be argued that this phospholipid did not originate from the nanoparticles themselves but rather from MVs which contaminated the nanoparticle samples. However, the nanoparticle samples obtained from cultures of *Rba. capsulatus* were only poorly contaminated by MVs (Fig. 1b). This suggests that the phospholipid identified in the MS-spectra of these particle samples is most likely associated with the Se^0^-nanoparticles. This assumption is consistent with results of other works reporting that insertion of hydrophobic nanoparticles into lipid bilayers is thermodynamically favorable [126, 127]. The tendency of hydrophobic nanoparticles to associate with bilayer membranes can lead to the formation of nanoparticle-vesicle hybrids [127], or to membrane-coated nanoparticles [128], depending on the size of the nanoparticles [126, 127], with nanoparticles larger than 6 nm being membrane-coated. We therefore propose that the Se^0^-nanoparticles produced intracellularly by *R. rubrum* and *Rba. capsulatus* are coated with components of the intracytoplasmic membrane, where the photochemical apparatus is concentrated [20], thus explaining association of the particles with BChl, BPhe, BPheide, as well as with intermediary metabolites of BChl biosynthesis and degradation products of these metabolites. Other components of the intracytoplasmic membrane should be associated with the particles as well. Consistently, the MS-spectra obtained from native particles embedded in the THAP-matrix (Additional file 3), and from organic particle extracts embedded in the DHB-matrix (Additional files 6 and 7), yielded many signals with masses corresponding to those expected for lipids (m/z 689.5, m/z 776.5, m/z 792.5), and, possibly, for quinone derivatives (m/z 835.5, and m/z 851.5).

On the other hand, the fact that intermediary metabolites of BChl biosynthesis together with degradation products of these metabolites were still present in detergent-washed particles, suggests that additional, so far unknown amphiphylic molecules, fill the space between the highly hydrophobic elemental selenium and the polar intermediary metabolites of BChl biosynthesis and degradation.

Consequently, further investigations are necessary for determining the actual composition of the Se^0^-nanoparticles excreted by purple phototrophic bacteria.

## Conclusion

MALDI-TOF spectrometry complemented with demetalation experiments has been proven useful for identifying small metabolites associated with Se^0^-nanoparticles produced by purple phototrophic bacteria grown in the presence of a toxic concentration of selenite. These methods allowed showing that BChl *a*, BPhe *a*, and BPheide *a*, which are known to accumulate in stationary phase cultures of purple phototrophic bacteria, were associated with the particles. Moreover, not only these compounds were identified in the particles, but also intermediary metabolites of the BChl biosynthesis pathway as well as a degradation product of both the demetalated protochlorophyllide (m/z 595.3) and the demetalated 3-acetoxy chloropyllide *a* (m/z 616.2), which correspond to the demetalated substrates of DPOR and COR, respectively. Accumulation of these various compounds was proposed to result from inactivation of many enzymes involved in BChl biosynthesis and, more precisely, from the degradation of [Fe-S]-clusters contained in the active site of these enzymes. Molecular oxygen and ROS, which are known to be produced during the reduction of selenite with glutathione, were proposed to be responsible of [Fe-S]-clusters and intermediary metabolite degradations. This proposition is consistent with results reported in previous works, indicating that cysteine desulfurase (IscS), an enzyme involved in [Fe-S] cluster repair, is largely induced in cultures of *E. coli* grown in the presence of a toxic concentration of selenite.

Accumulation of BChlide *a*, the substrate of BChl synthase, was also proposed to arise from damage of the cytoplasm-exposed substrate-binding site of this enzyme by ROS produced during selenite reduction, and by the reaction of selenite with the cysteine present in its substrate-binding site.

Altogether these results indicated that selenite toxicity significantly affects the function of the photosynthetic system of the purple phototrophic bacteria. They furthermore suggested that the Se^0^–nanoparticles produced by *R.rubrum* and *Rba. capsulatus* were coated with components of the intracytoplasmic membrane. This proposition is strengthened by MS-analysis of organic particle extracts showing that, in addition to BChl *a*, BPhe *a*, BPheide *a*, and intermediary metabolites of the BChl biosynthesis pathway, the particles were associated with many compounds yielding masses comprised between m/z 700 and m/z 850 which, according to their mass and to their solubility in organic solvents, were assumed to represent lipids and, possibly, quinone derivatives.

## Additional files

Additional file 1 Representative MS-spectra of the references BPhe *a*_p_ and BChl *a*_p_ prepared using the DHB-matrix. (PDF 185 kb)

Additional file 2 Representative MS-spectra of the references BPhe *a*_p_ and BChl *a*_p_ prepared using the THAP-matrix. (PDF 169 kb)

Additional file 3 Matrix effect on the MS-spectra of native Se^0^-nanoparticle samples isolated from cultures of *R. rubrum*. (PDF 116 kb)

Additional file 4 MS-spectra of detergent-washed Se^0^-nanoparticles. Effect of the washing procedure on the composition of the particle samples. (PDF 163 kb)

Additional file 5 Schematic representation of tandem MS-spectra of MS-signals at m/z 786.6, m/z 808.6 and m/z 824.6. (PDF 157 kb)

Additional file 6 MS-spectra of organic solvent extracts from Se^0^-nanoparticle samples obtained from cultures of *R. rubrum*. (PDF 202 kb)

Additional file 7 MS-spectra of organic solvent extracts from Se^0^-nanoparticle samples obtained from cultures of *Rba. capsulatus*. (PDF 189 kb)

Additional file 8 Tandem MS-spectrum of PPIX-di-O-CH_3_. (PDF 114 kb)

## Abbreviations

(B)Chl *a*: (bacterio)chlorophyll *a*
(B)Chlide *a*: (bacterio)chlorophyllide *a*
(B)Phe *a*: (bacterio)pheophytin *a*
(B)Pheide *a*: (bacterio)pheophorbide *a*
DHB: 2,5-dihydrobenzoic acid
DHPC: Diheptanoyl-phosphatidylcholine
MALDI: Matrix assisted laser desorption ionisation
MS: Mass spectrometry
MV: Membrane vesicle
PAO: Pheophorbide *a* oxygenase
PChlide: Protochlorophyllide
PPIX: Protoporphyrin IX
ROS: Reactive oxygen species
Se^0^: elemental selenium
SOD: Superoxide dismutase
TEM: Transmission electron microscopy
THAP: 2,4,6-trihydroxyacetophenone
