# Supplementary material for: Inhibition of bacteriochlorophyll biosynthesis in the purple phototrophic bacteria Rhodospirillumrubrum and Rhodobacter capsulatus grown in the presence of a toxic concentration of selenite
Source: BMC Microbiol. 2018 Jul 31;18:81. doi: 10.1186/s12866-018-1209-5 (PMC6069883; doi:10.1186/s12866-018-1209-5)
Supplement: Supplementary file 1 — Representative MS-spectra of the references BPhe ap and BChl ap prepared using the DHB-matrix. (PDF 185 kb) [file 12866_2018_1209_MOESM1_ESM.pdf]

Representative MS-spectra of the references BPhe a_p and BChl a_p prepared using the DHB-matrix.

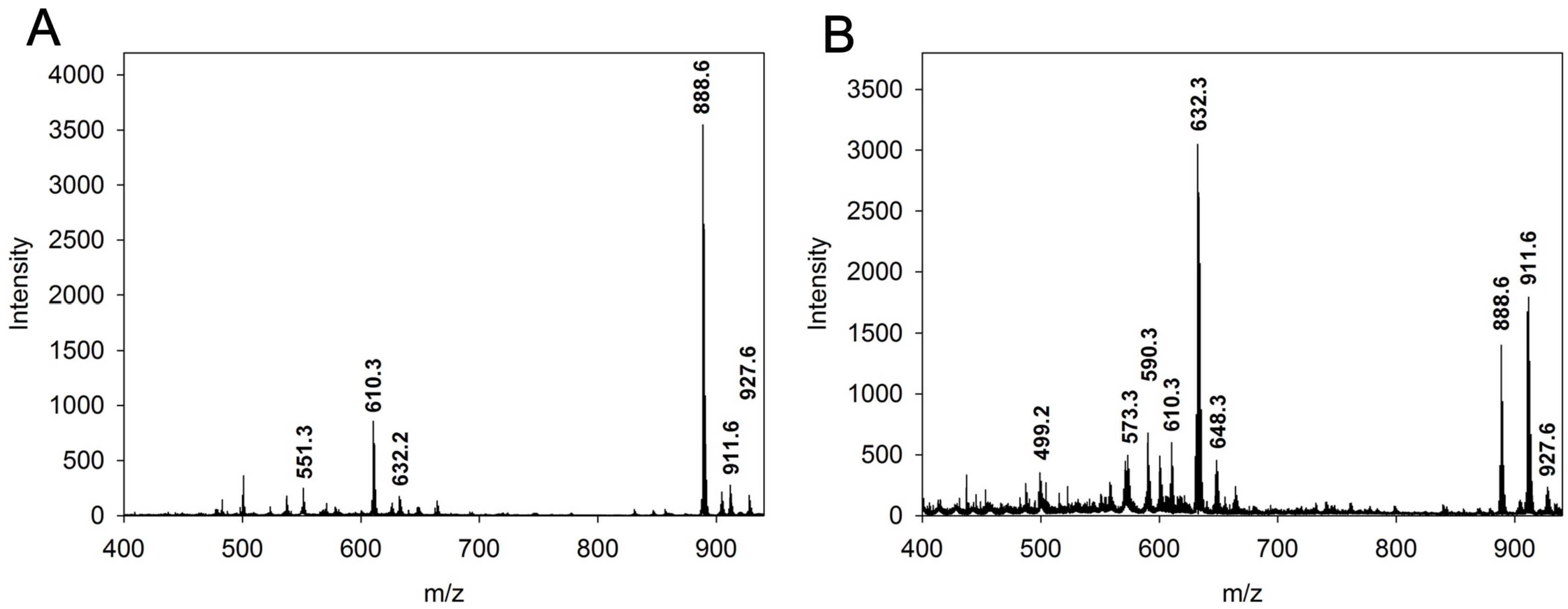

A: MS-spectrum of the reference BPhe a_p obtained by demetalation of the reference BChl a_p using conc. acetic acid. The signal of high intensity representing BPhe a_p was accompanied by low intensity signals for BChlide a (m/z 632.3), BPheide *a* (m/z 610.3) and pyro-BPheide *a* (m/z 551.3). As BPhe a_p was not purified after demetalation, these signals were considered to predominantly result from the demetalation process. Consequently, BPhe a_p prepared with the DHB-matrix was considered to be stable under laser radiation as reported previously [1].
B: MS-spectrum of the reference BChl a_p. The signal of relatively high intensity for BChl a_p (m/z 911.6) was accompanied by a signal of high intensity for BChlide *a* (m/z 632.3), a relatively intense signal for BPhe a_p (m/z 888.6) and signals of relatively low intensity for BPheide *a* (m/z 610.3), pyro-BChlide *a* (m/z 573.3), allomers of BChlide *a* (m/z 648.3) and of pyro-BChlide *a* (m/z 590.3), as well as for other degradation products, indicating that BChl a_p embedded in the DHB-matrix was significantly less stable under laser radiation compared to BPhe a_p.

Reference

1. Suzuki, T., Midonoya, H., Shioi, Y.: Analysis of chlorophylls and their derivatives by matrix-assisted laser desorption/ionization-time-of-flight mass spectrometry. Anal Biochem 390(1), 57{62 (2009)
