# Supplementary material for: Inhibition of bacteriochlorophyll biosynthesis in the purple phototrophic bacteria Rhodospirillumrubrum and Rhodobacter capsulatus grown in the presence of a toxic concentration of selenite
Source: BMC Microbiol. 2018 Jul 31;18:81. doi: 10.1186/s12866-018-1209-5 (PMC6069883; doi:10.1186/s12866-018-1209-5)
Supplement: Supplementary file 2 — Representative MS-spectra of the references BPhe ap and BChl ap prepared using the THAP-matrix. (PDF 169 kb) [file 12866_2018_1209_MOESM2_ESM.pdf]

# Representative MS-spectra of the references BPhe $a_p$ and BChl $a_p$ prepared using the THAP-matrix.

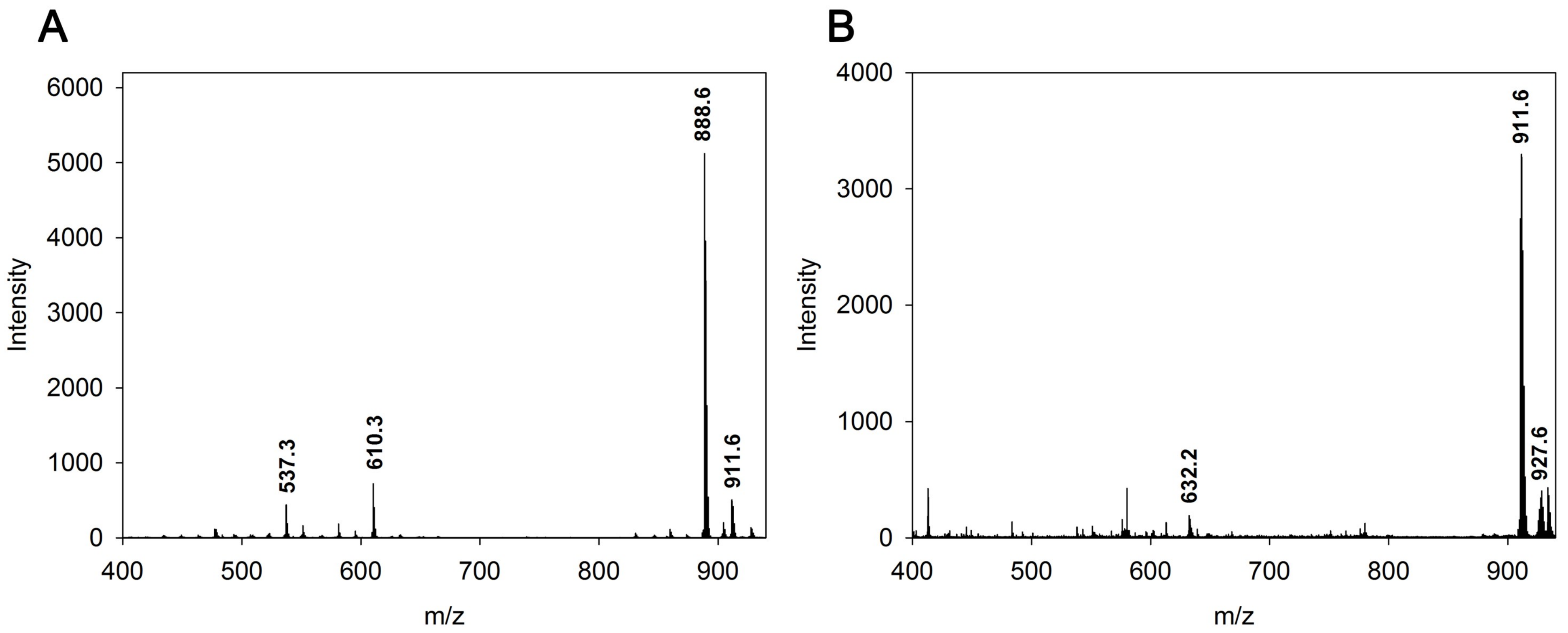


**A:** MS-spectrum of the reference BPhe $a_p$ obtained by demetalation of the reference BChl $a_p$. Signals of relatively low intensity were present for BPheide *a* at m/z 610.3, and for a degradation product of BPheide *a* at m/z 537.3. As already indicated in the legend to the Additional file 1 these signals likely resulted from the preparation of BPhe $a_p$, which was not purified after demetalation of BChl $a_p$ with conc. acetic acid. Other signals for degradation products showed low intensity, indicating that BPhe $a_p$ embedded in the THAP-matrix was well preserved under laser radiation.
**B:** MS-spectrum of BChl $a_p$. It showed only signals of low intensity for degradation products, indicating that Bchl $a_p$ was much better preserved from degradation under laser radiation when embedded in the THAP-matrix than in the DHB-matrix (see Additional File 1).
