# Supplementary material for: Inhibition of bacteriochlorophyll biosynthesis in the purple phototrophic bacteria Rhodospirillumrubrum and Rhodobacter capsulatus grown in the presence of a toxic concentration of selenite
Source: BMC Microbiol. 2018 Jul 31;18:81. doi: 10.1186/s12866-018-1209-5 (PMC6069883; doi:10.1186/s12866-018-1209-5)
Supplement: Supplementary file 3 — Matrix effect on the MS-spectra of native Se0-nanoparticle samples isolated from cultures of R. rubrum. (PDF 116 kb) [file 12866_2018_1209_MOESM3_ESM.pdf]

Matrix effect on the MS-spectra of native Se^0-nanoparticle samples isolated from cultures of *R. rubrum*.

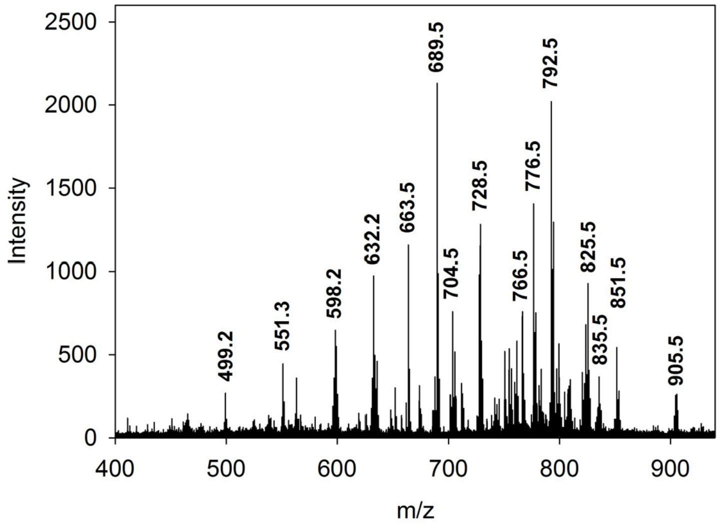

Samples embedded in the THAP-matrix.
By contrast to the MS-spectrum of these particle samples embedded in the DHB-matrix (Figure 3A), this MS-spectrum showed many unidentified signals, while signals for PPIX and PPIX-O-CH3 as well as for BPheide *a*, Mg-PPIX-O-CH3 and m/z 616.2 were not detected in samples embedded in this matrix. Consequently, the THAP-matrix could not be used for efficiently detecting the presence of molecules representing BChl *a* degradation products and intermediary metabolites of the BChl *a* biosynthesis contained in the Se^0-nanoparticle samples.
