# Supplementary material for: Inhibition of bacteriochlorophyll biosynthesis in the purple phototrophic bacteria Rhodospirillumrubrum and Rhodobacter capsulatus grown in the presence of a toxic concentration of selenite
Source: BMC Microbiol. 2018 Jul 31;18:81. doi: 10.1186/s12866-018-1209-5 (PMC6069883; doi:10.1186/s12866-018-1209-5)
Supplement: Supplementary file 4 — MS-spectra of detergent-washed Se0-nanoparticles. Effect of the washing procedure on the composition of the particle samples. (PDF 163 kb) [file 12866_2018_1209_MOESM4_ESM.pdf]

MS-spectra of detergent-washed Se^0 -nanoparticles. Effect of the washing procedure on the composition of the particle samples.

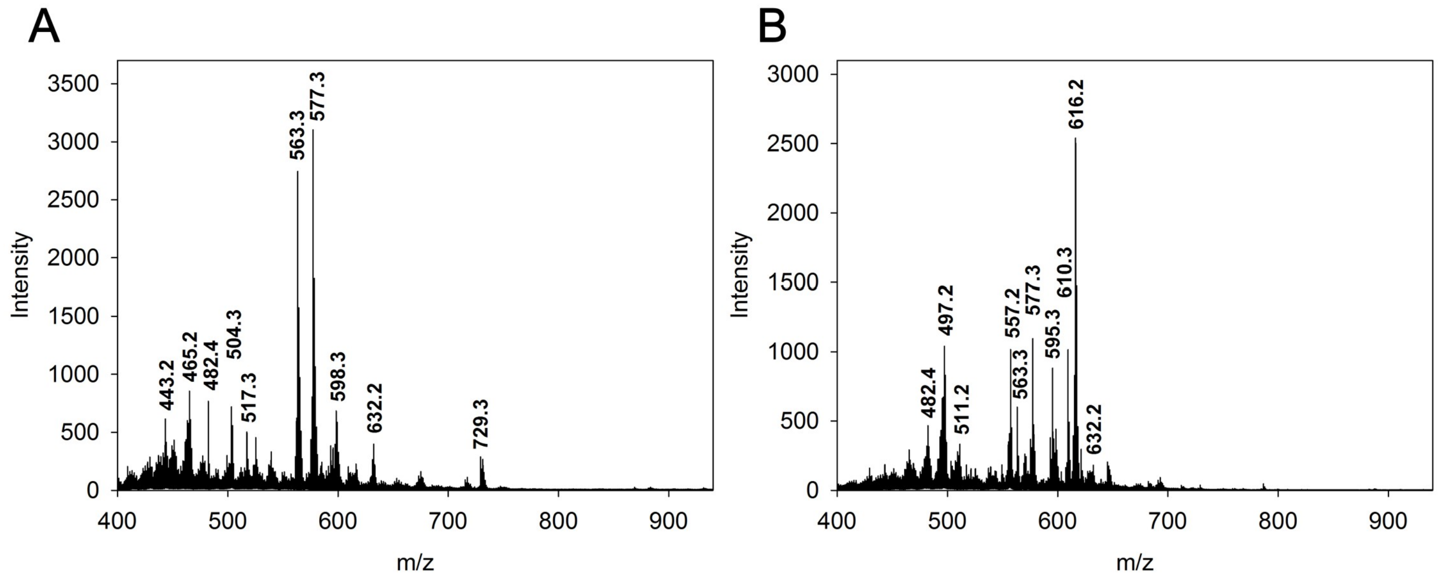

The samples were embedded in the DHB matrix.
A: Particle samples isolated from cultures of *R. rubrum*. **B:** Particle samples isolated from cultures of *Rba. capsulatus.*
In the MS-spectra of both particle species, the MS-signals assumed to represent hydrophobic molecules such as BChl a_p at m/z 911.5, BChl a_g at m/z 905.5, BPhe a_p at m/z 888.6, and BPhe a_g at m/z 882.6 (see Table 1) disappeared from the MS-spectra after the washing procedure. But many signals which were present in the MS-spectra of the native particle samples (see Figure 3) were still present in the MS-spectra of the detergent-washed particles, indicating that the corresponding compounds were tightly associated with the particles. As these molecules were attributed to PPIX at m/z 563.3, PPIX-O-CH3 at m/z 577.3, BPheide *a* at m/z 610.3, BChlide *a* at m/z 632.2, which represent relatively hydrophilic molecules, and as pure elemental selenium is highly hydrophobic, this result indicated that the composition of the Se^0-nanoparticles may be very complex (see Discussion). The signal at m/z 482.4 represented the detergent DHPC. For identification of the other signals see text. The molecule corresponding to the signal at m/z 729.3 (particles produced by *R. rubrum*) has not been identified.
