# Supplementary material for: Inhibition of bacteriochlorophyll biosynthesis in the purple phototrophic bacteria Rhodospirillumrubrum and Rhodobacter capsulatus grown in the presence of a toxic concentration of selenite
Source: BMC Microbiol. 2018 Jul 31;18:81. doi: 10.1186/s12866-018-1209-5 (PMC6069883; doi:10.1186/s12866-018-1209-5)
Supplement: Supplementary file 5 — Schematic representation of tandem MS-spectra of MS-signals at m/z 786.6, m/z 808.6 and m/z 824.6. (PDF 157 kb) [file 12866_2018_1209_MOESM5_ESM.pdf]

Schematic representation of tandem MS-spectra of MS-signals at m/z 786.6, m/z 808.6 and m/z 824.6.

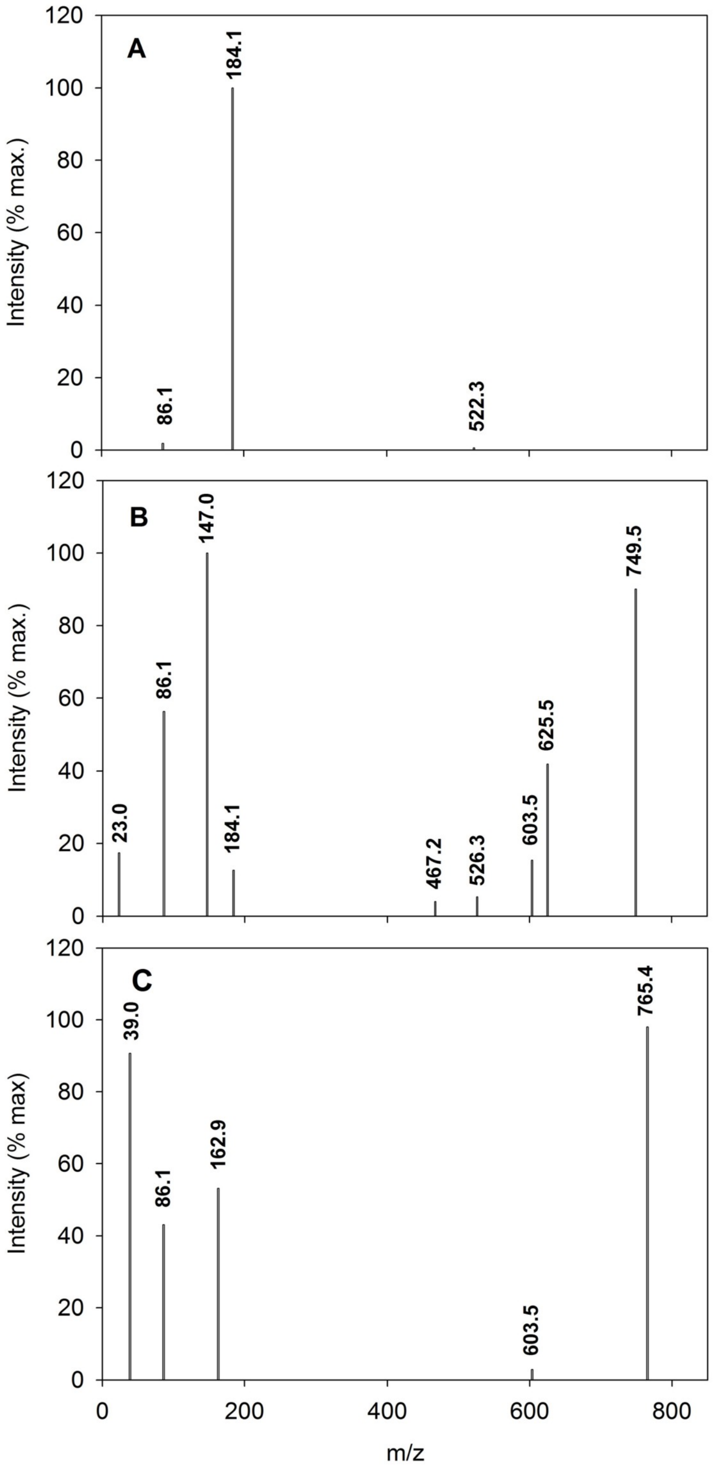

According to literature data [1] the tandem MS-spectrum of (A) m/z 786.6 typically represented a phosphatidyl-choline phospholipid. Analysis of the MS/MS-spectra of the signals in (B) m/z 808.6 and in (C) m/z 824.6 led to the conclusion that they represented the Na+ -adduct and the K+ -adduct, respectively, of the same phospholipid [1, 2]. These tandem MS-spectra allowed to identify m/z 786.6 as dioleoyl phosphatidylcholine. This result was consistent with the presence of phosphatidylcholine phospholipids in the membrane of *Rba. capsulatus* [3]. As the detectability of different lipid classes is dependent on the headgroup structure, and as phosphatidylcholine phospholipids are the most sensitively detectable lipids [4], we assume that other lipids, not identified in this work, are possibly associated with the Se^0 nanoparticle samples isolated from cultures of *R. rubrum* and *Rba. capsulatus*. Consistent with this proposition is the presence of signals with masses in the range of 650 to 850 mass units obtained in the organic solvent extracts of particle samples isolated from cultures of both bacterial species (Additional file 6 and Additional file 7). Further lipid analysis will, therefore, be required for determining the actual lipid composition of these particle samples. Consistent with their proposed lipid trait, these signals disappeared from the mass spectra of detergent washed Se^0-nanoparticles (Additional file 4).

References

1. Hsu, F.-F.u., Turk, J.: Electrospray ionization/tandem quadrupole mass spectrometric studies on phosphatidyl-cholines: the fragmentation processes. J Am Soc Mass Spectrom 14(4), 352{363 (2003)
2. Han, X., Gross, R.W.: Structural determination of picomole amounts of phospholipids via electrospray ionization tandem mass spectrometry. J Am Soc Mass Spectrom 6(12), 1202{1210 (1995)
3. Imhoff, J.F., Bias-Imho , U.: Lipids, Quinones and Fatty Acids of Anoxygenic Phototrophic Bacteria. In: Blanken-ship, R.E., Madigan, M.T., Bauer, C.E. (eds.) Anoxigenic Photosynthetic Bacteria. Advances in Photosynthesis and Respiration, vol. 2, pp. 179{205. Springer, Netherland (1995). Chap. 10
4. Schiller, J., Suss, R., Fuchs, B., Muller, M., Zschornig, O., Arnold, K.: MALDI-TOF MS in lipidomics. Front Biosci 12, 2568{2579 (2007)
