# Supplementary material for: Inhibition of bacteriochlorophyll biosynthesis in the purple phototrophic bacteria Rhodospirillumrubrum and Rhodobacter capsulatus grown in the presence of a toxic concentration of selenite
Source: BMC Microbiol. 2018 Jul 31;18:81. doi: 10.1186/s12866-018-1209-5 (PMC6069883; doi:10.1186/s12866-018-1209-5)
Supplement: Supplementary file 6 — MS-spectra of organic solvent extracts from Se0-nanoparticle samples obtained from cultures of R. rubrum. (PDF 202 kb) [file 12866_2018_1209_MOESM6_ESM.pdf]

MS-spectra of organic solvent extracts from Se^0-nanoparticle samples obtained from cultures of *R. rubrum*. Effect of acid treatment on the sample composition.

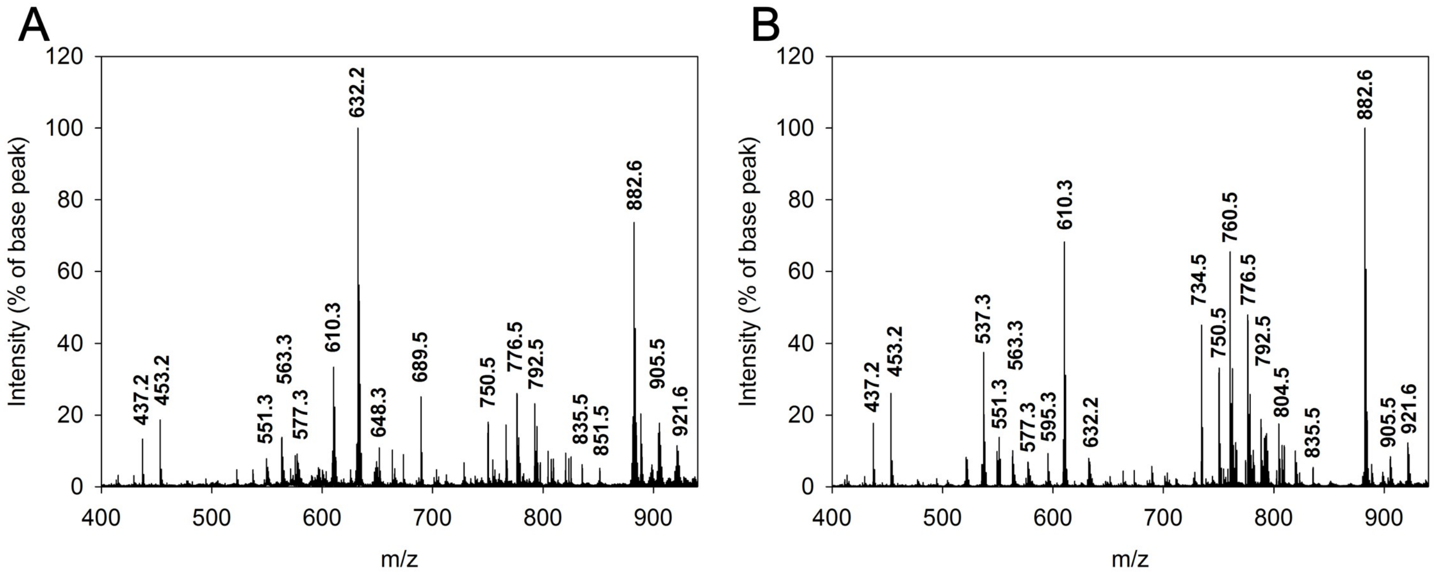

The samples were embedded in the DHB-matrix. Each figure represents the mean of two different spectra obtained from two different particle sample extracts.

A: Untreated extracts.

In this MS-spectrum BChlide *a* (m/z 632.2), yielded a signal of high intensity, indicating that this molecule was relatively well preserved from demetalation during the particle extraction process.

Surprisingly the signals for BChl a_g at m/z 905.5 and, particularly that for BPhe a_g at m/z 882.5, yielded signals of significantly higher intensities in the MS-spectra of particle extracts than in the MS-spectra of native particle samples (Fig. 3A). This result indicated that these compounds were more efficiently detected in particle extracts than in native particle samples. Low detection efficiency in native particle samples was attributed to interactions of these compounds with other components of the particles. The high intensity of the signal for BPhe a_g at m/z 882.5 obtained before acid treatment may indicate that BChl a_g was partially demetalated during the extraction process.

Note the appearance of MS-signals between m/z 650 and m/z 860 in the particle extract, which were absent from the MS-spectra of native particle samples (Fig. 3A). Consistent with their masses and their solubility in organic solvents the signals at m/z 689.5, m/z 750.5, m/z 776.5 and m/z 792.5 were assumed to represent lipids and the signals at m/z 835.5 and 851.5 were attributed to quinone derivatives. These signals were, however, not further analyzed.

B: Extracts treated with concentrated acetic acid.

The large decrease of the signal intensity for BChlide *a* at m/z 632.2, accompanied by a significant increase of the signal for BPheide *a* at m/z 610.3, showed that BChlide *a* was largely demetalated after acid treatment (see Table 2). Note the significant increase of the signal intensity for BPhe a_g at 882.6, accompanied by a decrease of the signal intensity for BChl a_g at m/z 905.5, indicating that, as expected, BChl a_g was further demetalated after acid treatment.

In this spectrum the signal intensity of the various signals with masses comprised between m/z 700 and m/z 800 also largely increased, suggesting that interactions of the corresponding molecules with other components of the particle extracts were lowered under strong acid condition.
