# Supplementary material for: Inhibition of bacteriochlorophyll biosynthesis in the purple phototrophic bacteria Rhodospirillumrubrum and Rhodobacter capsulatus grown in the presence of a toxic concentration of selenite
Source: BMC Microbiol. 2018 Jul 31;18:81. doi: 10.1186/s12866-018-1209-5 (PMC6069883; doi:10.1186/s12866-018-1209-5)
Supplement: Supplementary file 7 — MS-spectra of organic solvent extracts from Se0-nanoparticle samples obtained from cultures of Rba. capsulatus. (PDF 189 kb) [file 12866_2018_1209_MOESM7_ESM.pdf]

MS-spectra of organic solvent extracts from Se^0-nanoparticle samples obtained from cultures of *Rba. capsulatus*. Effect of acid treatment on the sample composition.

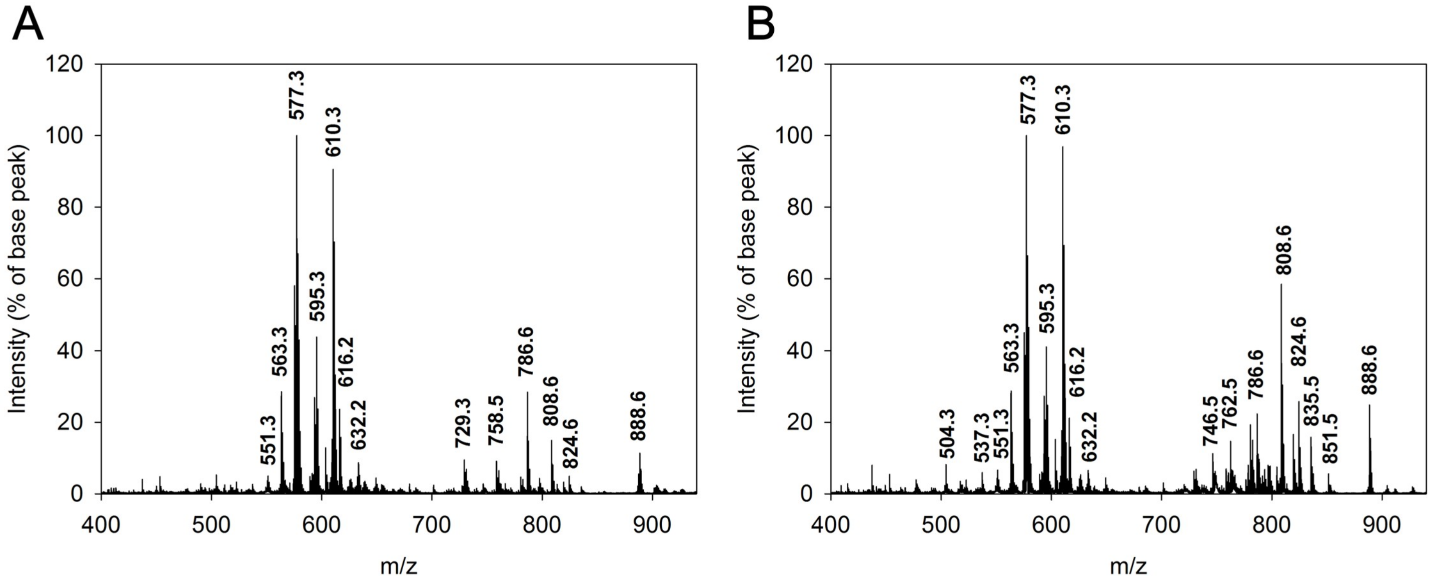

The samples were embedded in the DHB-matrix. Each figure represents the mean of two spectra obtained from two different sample extracts.
A: Untreated particle extracts.
In this MS-spectrum BChlide *a* (m/z 632.2) yielded a signal of much lower intensity and BPheide *a* (m/z 610.3) a signal of significantly higher intensity compared with the intensity of these signals obtained in the MS-spectrum of native particle samples (Fig. 3B), indicating that BChlide *a* was largely demetalated during the particle extraction process.
BPhe a_p, (m/z 888.6), yielded a signal of significantly higher intensity compared with that obtained in the MS-spectrum of native particle samples (Fig. 3) despite the fact that the signal for BChl a_p was extremely low in both the native particle samples and the particle sample extracts. This suggested that BChl a_p was not efficiently detected in neither the native particle samples nor in the particle sample extracts.
Note the presence of signals at m/z 729.3 and m/z 758.3, which were absent from the MS-spectra of the native particle samples. Their solubility in organic solvents indicated that they represent hydrophobic molecules.
B: Particle extracts treated with conc. acetic acid.
As demetalation was observed already in the MS-spectrum of untreated particle extracts, decrease of the signal intensity for BChlide *a* at m/z 632.2 and increase of BPheide *a* at m/z 610.3 were not significant after treatment with concentrated acetic acid.
The intensity of the signal for BPhe a_p (m/z 888.6) significantly increased after demetalation despite the fact that the intensity of the signal for BChl a_p was extremely low in the MS-spectra of both the native particle samples (Fig 3B) and the particle sample extracts. These results indicated once more that BChl a_p was not efficiently detected in both the native particle samples and the particle extracts.
Interestingly, the intensities of the signals at m/z 616.2 and m/z 595.3 were nearly not modified after acid treatment, confirming that the corresponding molecules were metal-free.
Note the appearance of new MS-signals between m/z 700 and m/z 860 after acid treatment, indicating that the corresponding compounds were better detected under strong than under moderate acid condition. According to their mass and their solubility in organic solvents the signals at m/z 746.5 and m/z 762.5 were assumed to represent lipids, and the signals at m/z 835.5 and m/z 851.5 were attributed to quinone derivatives.
