# Supplementary material for: Inhibition of bacteriochlorophyll biosynthesis in the purple phototrophic bacteria Rhodospirillumrubrum and Rhodobacter capsulatus grown in the presence of a toxic concentration of selenite
Source: BMC Microbiol. 2018 Jul 31;18:81. doi: 10.1186/s12866-018-1209-5 (PMC6069883; doi:10.1186/s12866-018-1209-5)
Supplement: Supplementary file 8 — Tandem MS-spectrum of PPIX-di-O-CH3. (PDF 114 kb) [file 12866_2018_1209_MOESM8_ESM.pdf]

Tandem MS-spectrum of PPIX-di-O-CH_3.

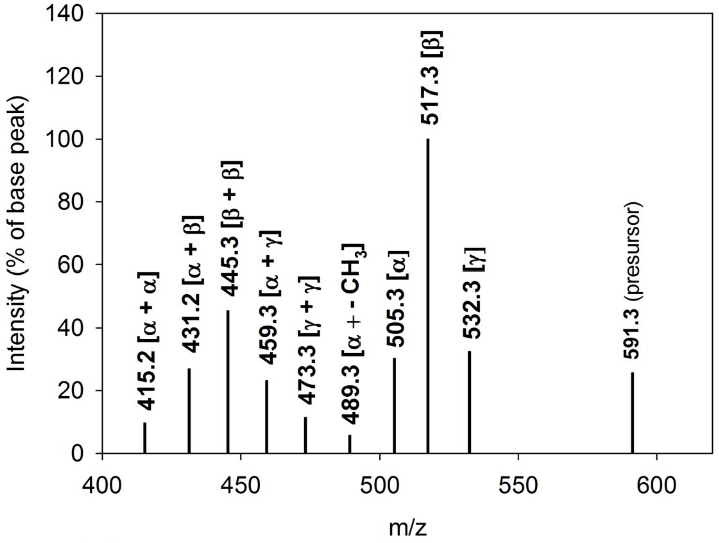

The base peak represents a molecular ion after the loss of a 74 mass units fragment (591.3 - 74 = 517.3), thus corresponding to the cleavage of a β-bond (see Figure 2). Signals of lower intensity represented the cleavages of α and γ bonds or combinations of α, β and γ bonds . These results were consistent with those reported by [1], indicating that the cleavages of the C13 and C17 substituents yield the most intense signals in tandem MS of porphyrin molecules, and that the base peak represented the cleavage of β-bound in these spectra as reported in previous works [2, 3].

References

1. Brockmann, J. Hans, Knobloch, G., Schweer, I., Trowitzsch, W.: Die Alkoholkomponente des Bacteriochlorophyll a aus Rhodospirillum rubrum. Arch Mikrobiol 90(2), 161-164 (1973)
2. Quirke, J.M.E.: Mass spectrometry of porphyrins and metalloporphyrins. In: Kadish, K.M., Smith, K.M., Guilard, R. (eds.) Theoretical and Physical Characterization. The Porphyrin Handbook, vol. 7, pp. 371-422. Academic Press, San Diego (2000). Chap. 54
3. Lim, C.K.: Mass Spectrometry of Porpohyrins. In: High-Performance Liquid Chromatography and Mass Spectrometry of Porphyrins, Chlorophylls and Bilins. Methods in chromatography, vol. 2, pp. 51-105. World Scientific, River Edge, N.J. (2010). Chap. 3
